# Structural Plasticity, Effectual Connectivity, and Memory in Cortex

**DOI:** 10.3389/fnana.2016.00063

**Published:** 2016-06-16

**Authors:** Andreas Knoblauch, Friedrich T. Sommer

**Affiliations:** ^1^Informatics Faculty, Albstadt-Sigmaringen UniversityAlbstadt, Germany; ^2^Redwood Center for Theoretical Neuroscience, University of California at BerkeleyBerkeley, CA, USA

**Keywords:** synaptic plasticity, effective connectivity, transfer entropy, learning, potential synapse, memory consolidation, storage capacity, spacing effect

## Abstract

Learning and memory is commonly attributed to the modification of synaptic strengths in neuronal networks. More recent experiments have also revealed a major role of structural plasticity including elimination and regeneration of synapses, growth and retraction of dendritic spines, and remodeling of axons and dendrites. Here we work out the idea that one likely function of structural plasticity is to increase “effectual connectivity” in order to improve the capacity of sparsely connected networks to store Hebbian cell assemblies that are supposed to represent memories. For this we define effectual connectivity as the fraction of synaptically linked neuron pairs within a cell assembly representing a memory. We show by theory and numerical simulation the close links between effectual connectivity and both information storage capacity of neural networks and effective connectivity as commonly employed in functional brain imaging and connectome analysis. Then, by applying our model to a recently proposed memory model, we can give improved estimates on the number of cell assemblies that can be stored in a cortical macrocolumn assuming realistic connectivity. Finally, we derive a simplified model of structural plasticity to enable large scale simulation of memory phenomena, and apply our model to link ongoing adult structural plasticity to recent behavioral data on the spacing effect of learning.

## 1. Introduction

Traditional theories attribute adult learning and memory to Hebbian modification of synaptic weights (Hebb, 1949; Bliss and Collingridge, 1993; Paulsen and Sejnowski, 2000; Song et al., 2000), whereas recent evidence suggests also a role for network rewiring by structural plasticity including generation of synapses, growth and retraction of spines, and remodeling of dendritic and axonal branches, both during development and adulthood (Raisman, 1969; Witte et al., 1996; Engert and Bonhoeffer, 1999; Chklovskii et al., 2004; Butz et al., 2009; Holtmaat and Svoboda, 2009; Xu et al., 2009; Yang et al., 2009; Fu and Zuo, 2011; Yu and Zuo, 2011). One possible function of structural plasticity is effective information storage, both in terms of space and energy requirements (Poirazi and Mel, 2001; Chklovskii et al., 2004; Knoblauch et al., 2010). Indeed, due to space and energy limitations, neural networks in the brain are only sparsely connected, even on a local scale (Abeles, 1991; Braitenberg and Schüz, 1991; Hellwig, 2000). Moreover, it is believed that the energy consumption of the brain is dominated by the number of postsynaptic potentials or, equivalently, the number of functional non-silent synapses (Attwell and Laughlin, 2001; Laughlin and Sejnowski, 2003; Lennie, 2003). Together this implies a pressure to minimize the number and density of functional (non-silent) synapses. It has therefore been suggested that the function of structural plasticity “moves” the rare expensive synapses to the most useful locations, while keeping the mean number of synapses on a constant low level (Knoblauch et al., 2014). By this, sparsely connected networks can have computational abilities that are equivalent to densely connected networks. For example, it is known that memory storage capacity of neural associative networks scales with the synaptic density, such that networks with a high connectivity can store many more memories than networks with a low connectivity (Buckingham and Willshaw, 1993; Bosch and Kurfess, 1998; Knoblauch, 2011). For modeling structural plasticity it is therefore necessary to define different types of “connectivity,” for example, to be able to distinguish between the actual number of anatomical synapses per neuron and the “potential” or “effectual” synapse number in an equivalent network with a fixed structure (Stepanyants et al., 2002; Knoblauch et al., 2014).

In this work we develop substantial new analytical results and insights focusing on the relation between network connectivity, structural plasticity, and memory. First, we work out the relation between “effectual connectivity” in structurally plastic networks and functional measures of brain connectivity such as “effective connectivity” and “transfer entropy.” Assuming a simple model of activity propagation between two cortical columns or areas, we argue that effectual connectivity is basically equivalent to the functional measures, while maintaining a precise anatomical interpretation. Second, we give improved estimates on the information storage capacity of a cortical macrocolumn as a function of effectual connectivity (cf., Stepanyants et al., 2002; Knoblauch et al., 2010, 2014). For this we develop exact methods (Knoblauch, 2008) to analyze associative memory in sparsely connected cortical networks storing random activity patterns by structural plasticity. Moreover, we generalize our analyses that are reasonable only for very sparse neural activity, to a recently proposed model of associative memory with structural plasticity (Knoblauch, 2009b, 2016) that is much more appropriate for moderately sparse activity deemed necessary to stabilize cell assemblies or synfire chains in networks with sparse connectivity (Latham and Nirenberg, 2004; Aviel et al., 2005). Third, we point out in more detail how effectual connectivity may relate to cognitive phenomena such as the spacing effect that learning improves if rehearsal is distributed to multiple sessions (Ebbinghaus, 1885; Crowder, 1976; Greene, 1989). For this, we analyze the temporal evolution of effectual connectivity and optimize the time gap between learning sessions to compare the results to recent behavioral data on the spacing effect (Cepeda et al., 2008).

## 2. Modeling

### 2.1. Memory, cell assemblies and synapse ensembles

*Memories* are commonly identified with patterns of neural activity that can be revisited, evoked and/or stabilized by appropriately modified synaptic connections (Hebb, 1949; Bliss and Collingridge, 1993; Martin et al., 2000; Paulsen and Sejnowski, 2000; for alternative views see Arshavsky, 2006). In the simplest case such a memory corresponds to a group of neurons that fire at the same time and, according to the Hebbian hypothesis that “what fires together wires together” (Hebb, 1949) develop strong mutual synaptic connections (Caporale and Dan, 2008; Clopath et al., 2010; Knoblauch et al., 2012). Such groups of strongly connected neurons are called *cell assemblies* (Hebb, 1949; Palm et al., 2014) and have a number of properties that suggest a function for associative memory (Willshaw et al., 1969; Marr, 1971; Palm, 1980; Hopfield, 1982; Knoblauch, 2011): For example, if a stimulus activates a subset of the cells, the mutual synaptic connections will quickly activate the whole cell assembly which is thought to correspond to the retrieval or completion of a memory. In a similar way, a cell assembly in one brain area *u* can activate an associated cell assembly in another brain area *v*. We call the set of synapses that supports retrieval of a given set of memories their *synapse ensemble S*. Memory consolidation is then the process of consolidating the synapses *S*.

**Figure 1:**
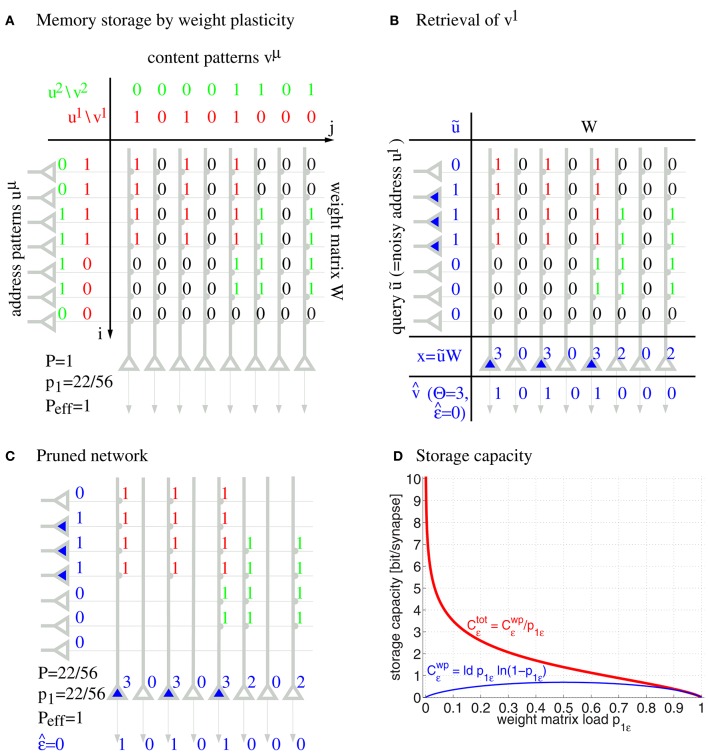
**Willshaw model for associative memory**. Panels show learning of two associations between activity patterns *u*^μ^ and *v*^μ^ **(A)**, retrieval of the first association **(B)**, pruning of irrelevant silent synapses **(C)**, and the asymptotic storage capacity in bit/synapse as a function of the fraction *p*_1_ of potentiated synapses **(D)** for networks with and without structural plasticity (*C*^tot^ vs. *C*^wp^; computed from Equations (49, 50, 47) for *P*_eff_ = 1; subscripts ϵ refer to maximized values at output noise level ϵ). Note that networks with structural plasticity can have a much higher storage capacity in sparsely potentiated networks with small fractions *p*_1_≪1 of potentiated synapses.

Formally, networks of cell assemblies can be modeled as associative networks, that is, single layer neural networks employing Hebbian-type learning. Figure 1 illustrates a simple associative network with clipped Hebbian learning (Willshaw et al., 1969; Palm, 1980; Knoblauch et al., 2010; Knoblauch, 2016) that associates binary activity patterns *u*^1^, *u*^2^, … and *v*^1^, *v*^2^, … within neuron populations *u* and *v* having size *m* = 7 and *n* = 8, respectively: Here synapses are binary, where a weight *W*_*ij*_ may increase from 0 to 1 if both presynaptic neuron *u*_*i*_ and postsynaptic neuron *v*_*j*_ have been synchronously activated for at least θ_*ij*_ times,

(1) $$\begin{array}{l} W_{ij}=\left\{ \begin{array}{l} \begin{array}{c} 1,\omega_{ij}\;:\text{​​}=\sum_{\mu =1}^{M}R(u_{i}^{\mu },v_{j}^{\mu })\;\geq \;\theta_{ij} \end{array}\\ 0,\text{ otherwise} \end{array}\right.. \end{array}$$

where *M* is the number of stored memories, ω_*ij*_ is called the synaptic potential, *R* defines a local learning rule, and θ_*ij*_ is the threshold of the synapse. In the following we will consider the special case of Equation (1) with Hebbian learning, $R\left(u_{i}^{\mu },v_{j}^{\mu }\right)=u_{i}^{\mu }\cdot v_{j}^{\mu }$, and minimal synaptic thresholds θ_*ij*_ = 1, which corresponds to the well-known Steinbuch or Willshaw model (Figure 1; cf., Steinbuch, 1961; Willshaw et al., 1969). Further, we will also investigate the recently proposed general “zip net” model, where both the learning rule *R* and synaptic thresholds θ_*ij*_ may be optimized for memory performance (Knoblauch, 2016): For *R* we assume the optimal homosynaptic or covariance rules, whereas synaptic thresholds θ_*ij*_ are chosen large enough such that the chance *p*_1_: = pr[*W*_*ij*_ = 1] of potentiating a given synapse is 0.5 to maximize entropy of synaptic weights (see Appendix A.3 for further details). In general, we can identify the synapse ensemble *S* that supports storage of a memory set 𝔐 by those neuron pairs *ij* with a sufficiently large synaptic potential ω_*ij*_≥θ_*ij*_ where θ_*ij*_ may depend on 𝔐. For convenience we may represent *S* as a binary matrix (with *S*_*ij*_ = 1 if *ij*∈*S* and *S*_*ij*_ = 0 if *ij*∉*S*) similar as the weight matrix *W*_*ij*_.

After learning a memory association *u*^μ^→*v*^μ^, a noisy input ũ can retrieve an associated memory content $\hat{v}$ in a single processing step by

(2) $$\begin{array}{l} {\hat{v}}_{j}=\left\{ \begin{array}{l} \begin{array}{c} 1,x_{j}=\left(\sum_{i=1}^{m}{\tilde{u}}_{i}W_{ij}+N_{j}\right)\geq \Theta_{j} \end{array}\\ 0,\text{ otherwise} \end{array}\right. \end{array}$$

for appropriately chosen neural firing thresholds Θ_*j*_. The model may include random variables N_*j*_ to account for additional synaptic inputs and further noise sources, but for most analyses and simulations (except Section 3.1) we assume N_*j*_ = 0 such that retrieval depends deterministically on the input ũ. In Figure 1B, stimulating with a noisy input pattern ũ ≈ *u*^1^ perfectly retrieves the corresponding output pattern $\hat{v}=v^{1}$ for thresholds Θ_*j*_ = 2. In the literature, input and output patterns are also called address and content patterns, and the (noisy) input pattern used for retrieval is called query pattern. In the illustrated completely connected network, the thresholds can simply be chosen according to the number of active units in the query pattern, whereas in biologically more realistic models, firing thresholds are thought to be controlled by recurrent inhibition, for example, regulating the number of active units to a desired level *l* being the mean activity of a content pattern (Knoblauch and Palm, 2001). Thus, a common threshold strategy in the more abstract models is to simply select the *l* most activated “winner” neurons having the largest dendritic potentials *x*_*j*_. In general, the retrieval outputs may have errors and the retrieval quality can then be judged by the output noise

(3) $$\begin{array}{l} \hat{\epsilon }=\frac{\sum_{j=1}^{n}|{\hat{v}}_{j}-v_{j}^{\mu }|}{l} \end{array}$$

defined as the Hamming distance between $\hat{v}$ and *v*^μ^ normalized to the mean number *l* of active units in an output pattern. Similarly, we can define input noise $\tilde{\epsilon }$ as the Hamming distance between ũ and *u*^μ^ normalized to the mean number *k* of active units in an input pattern.

In the illustrated network *u* and *v* are different neuron populations corresponding to hetero-association. However, all arguments will also apply to auto-association when *u* and *v* are identical (with *m* = *n*, *k* = *l*), and cell assemblies correspond to cliques of interconnected neurons. In that case output activity can be fed back to the input layer iteratively to improve retrieval results (Schwenker et al., 1996). Stable activation of a cell assembly can then expected if output noise $\hat{\epsilon }$ after the first retrieval step is lower than input noise $\tilde{\epsilon }$.

Capacity analyses show that each synapse can store a large amount of information. For example, even without any structural plasticity, the Willshaw model can store *C*^wp^ = 0.69 bit per synapse by weight plasticity (wp) corresponding to a large number of about *n*^2^/ log^2^ *n* small cell assemblies, quite close to the theoretical maximum of binary synapses (Willshaw et al., 1969; Palm, 1980). However, unlike in the illustration, real networks will not be fully connected, but, on a local scale of macrocolumns, the chance that two neurons are connected is only about 10% (Braitenberg and Schüz, 1991; Hellwig, 2000). In this case it is still possible to store a considerable number of memories, although maximal *M* scales with the number of synapses per neuron, and cell assemblies need to be relatively large in this case (Buckingham and Willshaw, 1993; Bosch and Kurfess, 1998; Knoblauch, 2011).

By including structural plasticity, for example, through pruning the unused silent synapses after learning in a network with high connectivity (Figure 1C), the total synaptic capacity of the Willshaw model can even increase to *C*^tot^ ~ log *n* ≫ 1 bit per (non-silent) synapse, depending on the fraction *p*_1_ of potentiated synapses (Figure 1D; see Knoblauch et al., 2010). Moreover, the same high capacity can be achieved for networks that are sparsely connected at any time, if the model includes ongoing structural plasticity and repeated memory rehearsal or additional consolidation mechanisms involving memory replay (Knoblauch et al., 2014).

In Section 3.2 we precisely compute the maximal number of cell assemblies that can be stored in a Willshaw-type cortical macrocolumn. As the Willshaw model is optimal only for extremely small cell assemblies with *k* ~ log *n* (Knoblauch, 2011), we will extend these results also for the general “zip model” of Equation (1) that performs close to optimal Bayesian learning even for much larger cell assemblies (Knoblauch, 2016).

### 2.2. Anatomical, potential, and effectual connectivity

**Figure 2:**
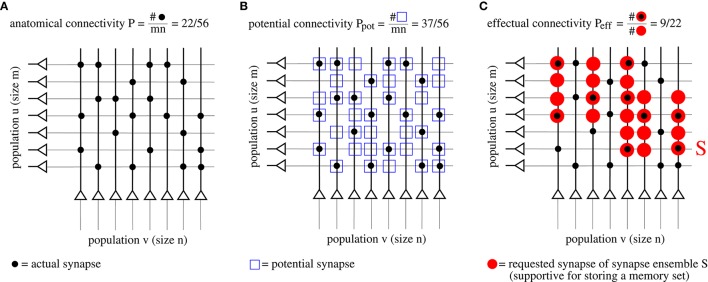
**Illustration of different types of “connectivity” corresponding to actual (A), potential (B), and requested synapses (C)**. The requested synapses in **(C)** correspond to the synapse ensemble *S* required to store the memory patterns in Figure 1.

As argued in the introduction, connectivity is an important parameter to judge performance. However, network models with structural plasticity need to consider different types of connectivity, in particular, anatomical connectivity *P*, potential connectivity *P*_pot_, effectual connectivity *P*_eff_, and target connectivity as measured by consolidation load *P*_1*S*_ (see Figure 2; cf., Krone et al., 1986; Braitenberg and Schüz, 1991; Hellwig, 2000; Stepanyants et al., 2002; Knoblauch et al., 2014),

(4) $$\begin{array}{l} P:=\frac{\text{\#actual synaptic connections}}{mn}\;, \end{array}$$

(5) $$\begin{array}{l} P_{\text{pot}}:=\frac{\text{\#potential synaptic connections}}{mn}, \end{array}$$

(6) $$\begin{array}{l} P_{\text{eff}}:=\frac{\sum_{i=1}^{m}\sum_{j=1}^{n}H\left(W_{ij}S_{ij}\right)}{\sum_{i=1}^{m}\sum_{j=1}^{n}H\left(S_{ij}^{2}\right)}\;, \end{array}$$

(7) $$\begin{array}{l} P_{1S}:=\frac{\sum_{i=1}^{m}\sum_{j=1}^{n}H\left(S_{ij}^{2}\right)}{mn}, \end{array}$$

where *H* is the Heaviside function (with *H*(*x*) = 1 if *x* > 0 and 0 otherwise) to include the general case of non-binary weights and synapse ensembles (*W*_*ij*_, *S*_*ij*_ ∈ ℝ).

First, *anatomical connectivity P* is defined as the chance that there is an actual synaptic connection between two randomly chosen neurons (Figure 2A)^1^. However, for example in the pruned network of Figure 1C, the anatomical connectivity *P* equals the fraction *p*_1_ of potentiated synapses (before pruning) and, thus, conveys only little information about the true (full) connectivity within a cell assembly. Instead, it is more adequate to consider potential and effectual connectivity (Figures 2B,C).

Second, *potential connectivity* *P*_pot_ is defined as the chance that there is a potential synapse between two randomly chosen neurons, where a potential synapse is defined as a cortical location *ij* where pre- and postsynaptic fibers are close enough such that a synapse could potentially be generated or has already been generated (Stepanyants et al., 2002).

Third, *effectual connectivity* *P*_eff_ defined as the fraction of “required synapses” that have already been realized is most interesting to judge the functional state of memories or cell assemblies during ongoing learning or consolidation with structural plasticity. Here we call the synapse ensemble *S*_*ij*_ required for stable storage of a given memory set also the *consolidation signal*. If *ij* corresponds to an actual synapse, we may identify the case *S*_*ij*_ > 0 with tagging synapse *ij* for consolidation (Frey and Morris, 1997). In case of simple binary network models such as the Willshaw or zip net models, the *S*_*ij*_ simply equal the optimal synaptic weights in a fully connected network after storing the whole memory set (Equation 1). Intuitively, if a set of cell assemblies or memories has a certain effectual connectivity *P*_eff_, then retrieval performance will be as if these memories would have been stored in a structurally static network with anatomical connectivity *P*_eff_, whereas true *P* in the structurally plastic network may be much lower than *P*_eff_.

Last, *target connectivity* or *consolidation load* *P*_1*S*_ is the fraction of neuron pairs *ij* that require a consolidated synapse as specified by *S*_*ij*_. This means that *P*_1*S*_ is a measure of the learning load of a consolidation task.

Note that our definitions of *P*_eff_ and *P*_1*S*_ apply as well to network models with gradual synapses (*W*_*ij*_, *S*_*ij*_ ∈ ℝ). More generally, by means of the consolidation signal *S*_*ij*_, we can abstract from any particular network model or application domain. Our theory is therefore not restricted to models of associative memory, but may be applied as well to other connectionist domains, given that the “required” synapse ensembles {*ij*|*S*_*ij*_≠0} and their weights can be defined properly by *S*_*ij*_. The following provides a minimal model to simulate the dynamics of effectual connectivity during consolidation.

### 2.3. Modeling and efficient simulation of structural plasticity

**Figure 3:**
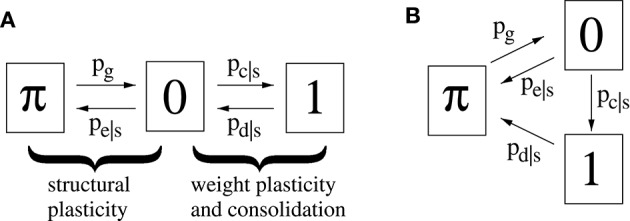
**Two simple models (A,B) of a potential synapses that can be used for simulating ongoing structural plasticity**. State π corresponds to potential but not yet realized synapses. State 0 corresponds to unstable silent synapses not yet potentiated or consolidated. State 1 corresponds to potentiated and consolidated synapses. Transition probabilities of actual synapses (state 0 or 1) depend on a consolidation signal *s* = *S*_*ij*_ that may be identified with the synaptic tags (Frey and Morris, 1997) marking synapses required to be consolidated for long-term memory storage. Thus, typically *p*_*c*|1_ > *p*_*c*|0_ for synaptic consolidation 0 → 1 and *p*_*e*|1_ < *p*_*e*|0_, *p*_*d*|1_ < *p*_*d*|0_ for synaptic elimination 0 → π and deconsolidation 1 → 0. All simulations assume synaptogenesis π → 1 (by *p*_*g*_) in homeostatic balance with synaptic elimination such that network connectivity *P* is constant over time.

Figure 3A illustrates a minimal model of a “potential” synapse that can be used to simulate the dynamics of ongoing structural plasticity (Knoblauch, 2009a; Deger et al., 2012; Knoblauch et al., 2014). Here a potential synapse *ij*^ν^ is the possible location of a real synapse connecting neuron *i* to neuron *j*, for example, a cortical location where axonal and dendritic branches of neurons *i* and *j* are close enough to allow the formation of a novel connection by spine growth and synaptogenesis (Krone et al., 1986; Stepanyants et al., 2002). Note that there may be multiple potential synapses per neuron pair, ν = 1, 2, …. The model assumes that a synapse can be either potential but not yet realized (state π), realized but still silent (state and weight 0), or realized and consolidated (state and weight 1).

For real synapses, state transitions are modulated by the consolidation signal *S*_*ij*_ specifying synapses to be potentiated and consolidated Then *structural plasticity* means the transition processes between states π and 0 described by transition probabilities *p*_*g*_: = pr[state(*t*+1) = 0|state(*t*) = π] and *p*_*e*|*s*_: = pr[state(*t*+1) = π|state(*t*) = 0, *S*_*ij*_ = *s*]. Similarly, *weight plasticity* means the transitions between states 0 and 1 described by probabilities *p*_*c*|*s*_: = pr[state(*t*+1) = 1|state(*t*) = 0, *S*_*ij*_ = *s*] and *p*_*d*|*s*_: = pr[state(*t*+1) = 0|*state*(*t*) = 1, *S*_*ij*_ = *s*]. For simplicity, we do not distinguish between long-term potentiation (LTP) and synaptic consolidation (or L-LTP), both corresponding to the transition from state 0 to 1. In accordance with the state diagram of Figure 3A, the evolution of synaptic states can then be described by probabilities $p_{\text{state}}^{\left(s\right)}\left(t\right)$ that a given potential synapse receiving *S*_*ij*_ = *s* is in a certain *state* ∈ {π, 0, 1} at time step *t* = 0, 1, 2, …,

(8) $$\begin{array}{l} p_{1}^{\left(s\right)}\left(t\right)=\;\left(1-p_{d|s\left(t\right)}\right)p_{1}^{\left(s\right)}\left(t-1\right)+p_{c|s\left(t\right)}p_{0}^{\left(s\right)}\left(t-1\right) \end{array}$$

(9) $$\begin{array}{l} p_{0}^{\left(s\right)}\left(t\right)=\;\left(1-p_{c|s\left(t\right)}-p_{e|s\left(t\right)}\right)p_{0}^{\left(s\right)}\left(t-1\right)+p_{d|s\left(t\right)}p_{1}^{\left(s\right)}\left(t-1\right)+\\ \;p_{g}p_{\pi }^{\left(s\right)}\left(t-1\right) \end{array}$$

(10) $$\begin{array}{l} p_{\pi }^{\left(s\right)}\left(t\right)=\;\left(1-p_{g}\right)p_{\pi }^{\left(s\right)}\left(t-1\right)+p_{e|s\left(t\right)}p_{0}^{\left(s\right)}\left(t-1\right)\\ \;=\;1-p_{1}^{\left(s\right)}\left(t\right)-p_{0}^{\left(s\right)}\left(t\right)\;, \end{array}$$

where the consolidation signal *s*(*t*) = *S*_*ij*_(*t*) may depend on time.

The second model variant (Figure 3B) can be described in a similar way except that *p*_*d*|*s*_ describes the transition from state 1 to state π. Model B is more convenient to analyze the spacing effect. We will see that, in relevant parameter ranges, both model variants behave qualitatively and quantitatively very similar. However, in most simulations we have used model A.

**Figure 4:**
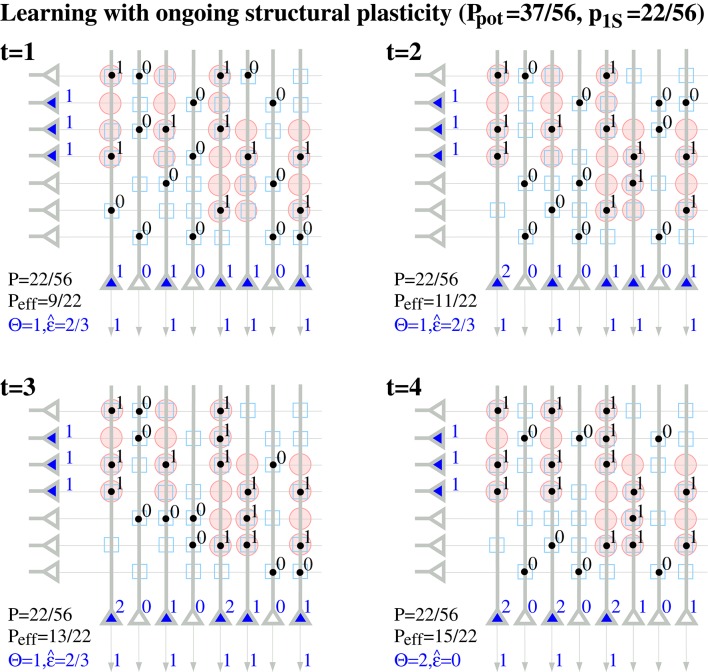
**Ongoing structural plasticity maintaining a constant anatomical connectivity ***P*** = 22/56 for the memory patterns of Figure 1 with actual, potential and requested synapses as in Figure 2 and assuming only single potential synapses per neuron pair (***p***(1) = 1, ***p***_***e***|***s***_ = 1−***s***, ***p***_***c***|***s***_ = ***s***, ***p***_***d***|***s***_ = 0)**. Note that *P*_eff_ increases with time from the anatomical level *P*_eff_ = 9/22 ≈ *P* at *t* = 1 toward the level of potential connectivity with *P*_eff_ = 15/22 ≈ *P*_pot_ at *t* = 4. Correspondingly, output noise $\hat{\epsilon }$ decreases with increasing *P*_eff_. At each time firing threshold Θ is chosen maximally to activate at least *l* = 3 neurons corresponding to the mean cell assembly size in the output population.

Note that a binary synapse in the original Willshaw model (Equation 1, Figures 1A,B) is a special case of the described potential synapse (*p*_*g*_ = *p*_*e*|*s*_ = *p*_*d*|*s*_ = 0, *p*_*c*|*s*_ = *s* ∈ {0, 1}, *S*_*ij*_ = *W*_*ij*_ as in Equation 1). Then pruning following a (developmental) learning phase (Figure 1C) can be modeled by the same parameters except increasing *p*_*e*|*s*_ > 0 to positive values. Finally, adult learning with ongoing structural plasticity can be modeled by introducing a homeostatic constraint to keep *P* constant (cf., Equation 69 in Appendix B.1; cf., Knoblauch et al., 2014), such that in each step the number of generated and eliminated synapses are about the same. Figure 4 illustrates such a simulation for *p*_*e*|*s*_ = 1−*s* and a fixed consolidation signal *S*_*ij*_ corresponding to the same memories as in Figure 1. Here the instable silent (state 0) synapses take part in synaptic turnover until they grow at a tagged location *ij* with *S*_*ij*_ = 1 where they get consolidated (state 1) and escape further turnover. This process of increasing effectual connectivity (see Equation 70 in Appendix B.2) continues until all potential synapses with *S*_*ij*_ = 1 have been realized and consolidated (Figure 4, *t* = 4) or synaptic turnover comes to an end if all silent synapses have been depleted.

Microscopic simulation of large networks of potential synapses can be expensive. We have therefore developed a method for efficient simulation of structural plasticity on a macroscopic level: Instead of the lower case probabilities (Equations 8–10) we consider additional memory-specific upper-case connectivity variables $P_{\text{state}}^{\left(s\right)}$ defined as the fractions of neuron pairs *ij* that receive a certain consolidation signal *s*(*t*) = *S*_*ij*_(*t*) and are in a certain state ∈ {∅, π, 0, 1} (where ∅ denotes neuron pairs without any potential synapses). In general it is

(11) $$\begin{array}{l} P_{1}^{\left(s\right)}\left(t\right)=P_{\text{pot}}^{\left(s\right)}\overset{\infty }{\underset{𝔫=1}{\sum }}𝔭\left(𝔫\right)\left(1-{\left(1-p_{1}^{\left(s\right)}\left(t\right)\right)}^{𝔫}\right) \end{array}$$

(12) $$\begin{array}{l} P_{\pi }^{\left(s\right)}\left(t\right)=P_{\text{pot}}^{\left(s\right)}\overset{\infty }{\underset{𝔫=1}{\sum }}𝔭\left(𝔫\right){\left(p_{\pi }^{\left(s\right)}\left(t\right)\right)}^{𝔫} \end{array}$$

(13) $$\begin{array}{l} P_{0}^{\left(s\right)}\left(t\right)=P_{\text{pot}}^{\left(s\right)}-P_{1}^{\left(s\right)}\left(t\right)-P_{\pi }^{\left(s\right)}\left(t\right) \end{array}$$

where $p_{1}^{\left(s\right)}$ and $p_{\pi }^{\left(s\right)}$ are as in Equations (8, 10); $P_{\text{pot}}^{\left(s\right)}$ is the fraction of neuron pairs receiving *s* that have at least one potential synapse; and 𝔭(𝔫) is the conditional distribution of potential synapse number 𝔫 per neuron pair having at least one potential synapse. Thus, we define a pre-/postsynaptic neuron pair *ij* to be in state 1 iff it has at least one state-1 synapse; in state 0 iff it does not have a state-1 synapse but at least one state-0 synapse; and in state π if it is neither in state 1 nor state 0 but has at least one potential synapse. See Fares and Stepanyants (2009) for neuroanatomical estimates of 𝔭(𝔫) in various cortical areas.

Summing over *s* we obtain further connectivity variables *P*_1_, *P*_0_, *P*_π_ from which we can finally determine the familiar network connectivities defined in the previous section,

(14) $$\begin{array}{l} P_{\text{state}}\left(t\right)=\underset{s}{\sum }P_{\text{state}}^{\left(s\right)}\left(t\right)\text{ for state}\in \left\{\emptyset ,\pi ,0,1\right\} \end{array}$$

(15) $$\begin{array}{l} P\left(t\right)=P_{0}\left(t\right)+P_{1}\left(t\right) \end{array}$$

(16) $$\begin{array}{l} P_{\text{pot}}\left(t\right)=P_{\pi }\left(t\right)+P_{0}\left(t\right)+P_{1}\left(t\right) \end{array}$$

(17) $$\begin{array}{l} P_{1S}=\underset{s\neq 0}{\sum }\underset{\text{state}\in \left\{\emptyset ,\pi ,0,1\right\}}{\sum }P_{\text{state}}^{\left(s\right)}\left(t\right) \end{array}$$

(18) $$\begin{array}{l} P_{\text{eff}}(t)=\frac{\sum_{s\neq 0}P_{1}^{\left(s\right)}(t)}{P_{1S}}\;. \end{array}$$

In general, the consolidation signal *s* = *s*(*t*) = *S*_*ij*_(*t*) will not be constant but may be a time-varying signal (e.g., if different memory sets are consolidated at different times). To efficiently simulate a large network of many potential synapses, we can partition the set of potential synapses in groups that receive the same signal *s*(*t*). For each group we can calculate the temporal evolution of state probabilities $p_{\pi }^{\left(s\right)}\left(t\right)$, $p_{0}^{\left(s\right)}\left(t\right)$, $p_{1}^{\left(s\right)}\left(t\right)$ of individual synapses from Equations (8–10). From this we can then compute from Equations (11–13) the group-specific macroscopic connectivity variables $P_{\pi }^{\left(s\right)}\left(t\right)$, $P_{0}^{\left(s\right)}\left(t\right)$, $P_{1}^{\left(s\right)}\left(t\right)$, and finally from Equations (14–18) the temporal evolution of the various network connectivities *P*_π_(*t*), *P*_0_(*t*), *P*_1_(*t*), *P*(*t*) as well as effectual connectivity *P*_eff_(*t*) for certain memory sets. For such an approach the computational cost of simulating structural plasticity scales only with the number of different groups corresponding to different consolidation signals *s*(*t*) (instead of the number of potential synapses as for the microscopic simulations).

Moreover, this approach is the basis for further simplifications and the analysis of cognitive phenomena like the spacing effect described in Appendix B. For example, for simplicity, the following simulations and analyses assume that each neuron pair *ij* can have at most a single potential synapse [i.e., 𝔭(1) = 1]. In previous works we have simulated also a model variant allowing multiple synapses per neuron pair, where we observed very similar results as for single synapses (Knoblauch et al., 2014). As synapse number per connected neuron pair has sometimes been reported to be narrowly distributed around a small number (e.g., 𝔫 = 4; cf., Fares and Stepanyants, 2009), one may also identify each single synapse in our model with a group of about 4 real cortical synapses (see Section 4).

This assumption is actually justified by evidence that 𝔫 is narrowly distributed around a small number, e.g., 𝔫 = 4 (Fares and Stepanyants, 2009). This means that two neurons are either unconnected or connected by a group of about four synapses (which is actually a very surprising finding as it is unclear how the neurons can regulate 𝔫; cf., Deger et al., 2012). This situation is well consistent with our modeling assumption 𝔭(1) = 1 if we identify each model synapse with such a group of about 4 real synapses.

## 3. Results

### 3.1. Information storage capacity, effectual connectivity and its relation to functional measures of brain connectivity

**Figure 5:**
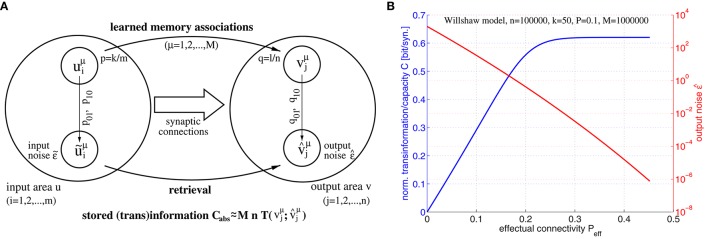
**Relation between effectual connectivity ***P***_**eff**_, information storage capacity ***C***, and output noise $\hat{\epsilon }$**. **(A)** Processing model for computing storage capacity *C*: = *C*_*abs*_/*Pmn* for *M* given memory associations between input patterns *u*^μ^ and output patterns *v*^μ^ stored in the synaptic weights (Equation 1; *p*: = pr[*u*^μ^ = 1], *q*: = pr[*v*^μ^ = 1]; *k* and *l* are mean cell assembly sizes in neuron populations *u* and *v*). During retrieval noisy address inputs ũ^μ^ with component errors $p_{ab}\;:=\text{pr}\left[{ũ}_{i}^{\mu }=b|u_{i}^{\mu }=a\right]$ and input noise $\tilde{\epsilon }\;:=p_{10}+\left(1/q-1\right)p_{01}$ are propagated through the network (Equation 2) yielding output patterns ${\hat{v}}^{\mu }$ with component errors $q_{ab}\;:=\text{pr}\left[{\hat{v}}_{j}^{\mu }=b|v_{j}^{\mu }=a\right]$ and output noise $\hat{\epsilon }=q_{10}+\left(1/q-1\right)q_{01}$. The retrieved information is then the transinformation between *v*^μ^ and ${\hat{v}}^{\mu }$. To simplify analysis, we assume independent transmission of individual (i.i.d.) memory bits $v_{j}^{\mu }$ over a binary channel with transmission errors *q*_01_, *q*_10_. **(B)** Information storage capacity *C*(*P*_eff_) (blue curve), and output noise $\hat{\epsilon }\left(P_{\text{eff}}\right)$ (red curve) as functions of effectual connectivity *P*_eff_ for a structurally plastic Willshaw network (similar to Figure 4) of *m* = *n* = 100, 000 neurons storing *M* = 10^6^ cell assemblies of sizes *k* = *l* = 50 and having anatomical connectivity *P* = 0.1 assuming zero input noise ($\tilde{\epsilon }=0$). Data have been computed similar to Equation (37) using Equations (44–46) for 0 ≤ *P*_eff_ ≤ *P*/*p*_1_.

For an information-theoretic evaluation, associative memories are typically viewed as memory channels that transmit the original content patterns *v*^μ^ and retrieve corresponding retrieval output pattern ${\hat{v}}^{\mu }$ (see Figure 5A). Thus, the absolute amount of transmitted or stored information *C*_*abs*_ of all *M* memories equals the transinformation or mutual information (Shannon and Weaver, 1949; Cover and Thomas, 1991)

(19) $$\begin{array}{l} C_{\text{abs}}:=T\left(\hat{V};V\right)\;:=\sum p\left(\hat{V},V\right)\log_{2}\frac{p\left(\hat{V},V\right)}{p\left(\hat{V}\right)\cdot p\left(V\right)} \end{array}$$

where *V*: = (*v*^1^, *v*^2^, …*v*^*M*^) and $\hat{V}\;:$ = $\left({\hat{v}}^{1},{\hat{v}}^{2},\ldots ,{\hat{v}}^{M}\right)$ correspond to the sets of original and retrieved content patterns, and *p*(.) to their probability distributions. If all *M* memories and *n* neurons have independent and identically distributed (i.i.d) activities (e.g., same fraction *q* of active units per pattern and component transmission error probabilities *q*_01_, *q*_10_), we can approximate this memory channel by a simple binary channel transmitting *M*·*n* memory bits $v_{j}^{\mu }\mapsto {\hat{v}}_{j}^{\mu }$ as assumed in Appendix A. Then

(20) $$\begin{array}{l} C_{\text{abs}}\approx M\cdot T\left({\hat{v}}^{\mu };v^{\mu }\right)\approx M\cdot n\cdot T\left(q,q_{01},q_{10}\right) \end{array}$$

where $T\left({\hat{v}}^{\mu };v^{\mu }\right)$ is the transinformation for single memory patterns and *T*(*q, q*_01_, *q*_10_) is the transinformation of a single bit (see Equation 38). From this we obtain the normalized information storage capacity *C* per synapse after dividing *C*_*abs*_ by the number of synapses *Pmn* (similar to Equation 37).

In our first experiment we have investigated the relation between information storage capacity and effectual connectivity *P*_eff_ during ongoing structural plasticity. For this we have assumed a larger network of size *m* = *n* = 100000 with anatomical connectivity *P* = 0.1 and larger cell assemblies with sizes *k* = *l* = 50, but otherwise a similar setting as for the toy example illustrated by Figure 4. Figure 5B shows output noise $\hat{\epsilon }$ and normalized capacity *C* as functions of effectual connectivity *P*_eff_ for a given number of *M* = 10^6^ random memories. Interestingly, both $\hat{\epsilon }$ and *C* turn out to be monotonic functions of *P*_eff_ because output errors decrease with increasing *P*_eff_ (see Equations 45, 46). Therefore, also output noise $\hat{\epsilon }\left(P_{\text{eff}}\right)$ decreases with increasing *P*_eff_ whereas, correspondingly, stored information per synapse *C*(*P*_eff_) increases with *P*_eff_. Because monotonic functions are invertible, we can thus conclude that effectual connectivity *P*_eff_ is an equivalent measure of information storage capacity or the transinformation (= mutual information) between the activity patterns of two neuron populations *u* and *v*. As can be seen from our data, *C*(*P*_eff_) tends to be even linear over a large range, *C*~*P*_eff_, until saturation occurs if $\hat{\epsilon }\to 0$ approaches zero corresponding to high-fidelity retrieval outputs.

Next, based on the this equivalence between *P*_eff_ and *C*, we work out the close relationship between *P*_eff_ and commonly used functional measures of brain connectivity. Recall that we have introduced “effectual connectivity” as a measure of memory related synaptic connectivity (Figure 2C) that shares with other definitions of connectivity (such as anatomical and potential connectivity) the idea that any “connectivity” measure should correspond to the chance of finding a connection element (such as an actual or potential synapse) between two cells. By contrast, in brain imaging and connectome analysis (Friston, 1994; Sporns, 2007) the term “connectivity” has a more heterogeneous meaning ranging from patterns of synaptic connections (anatomical connectivity) and correlations between neural activity (functional connectivity) to causal interactions between brain areas. The latter is also referred to as “effective connectivity” although usually measured in information theoretic terms (bits) such as delayed mutual information or transfer entropy (Schreiber, 2000). For example, in the simplest case the transfer entropy between activities *u*(*t*) and *v*(*t*) measured in two brain areas *u* and *v* is defined as

(21) $$\begin{array}{l} T_{u\to v}\;:=\sum p\left(v\left(t+1\right),u\left(t\right),v\left(t\right)\right)\log_{2}\frac{p\left(v\left(t+1\right)|u\left(t\right),v\left(t\right)\right)}{p\left(v\left(t+1\right)|v\left(t\right)\right)} \end{array}$$

where *p*(.) denotes the distribution of activity patterns (see Equation 4 in Schreiber, 2000)^2^. Such ideas of effective connectivity come from the desire to extract directions of information flow between two brain areas from measured neural activity, contrasting with (symmetric) correlation measures that can neither detect processing directions nor distinguish between causal interactions and correlated activity due to a common cause.

To see the relation between these functional measures of “effective connectivity” and *P*_eff_, first, note that transfer entropy equals the well-known conditional transinformation or conditional mutual information between *v*(*t*+1) and *u*(*t*) given *v*(*t*) (Dobrushin, 1959; Wyner, 1978),

(22) $$\begin{array}{l} T\left(v\left(t+1\right);u\left(t\right)|v\left(t\right)\right)\;:=\\ \;\sum p\left(v\left(t+1\right),u\left(t\right),v\left(t\right)\right)\log_{2}\frac{p\left(v\left(t+1\right),u\left(t\right)|v\left(t\right)\right)}{p\left(v\left(t+1\right)|v\left(t\right)\right)\cdot p\left(u\left(t\right)|v\left(t\right)\right)} \end{array}$$

(23) $$\begin{array}{l} =\sum p\left(v\left(t+1\right),\;u\left(t\right),v\left(t\right)\right)\;\log_{2}\\ \frac{p\left(v\left(t+1\right)|u\left(t\right),v\left(t\right)\right)\cdot p\left(u\left(t\right)|v\left(t\right)\right)}{p\left(v\left(t+1\right)|v\left(t\right)\right)\cdot p\left(u\left(t\right)|v\left(t\right)\right)}=T_{u\to v}\;. \end{array}$$

Second, we may apply this to one-step retrieval in an associative memory (Equation 2). Then *u*(*t*) = ũ^μ^ is a noisy input, and the update $v\left(t+1\right)=F\left(u\left(t\right)\right)={\hat{v}}^{\mu }$ produces the corresponding output pattern, where the mapping *F* corresponds to activity propagation through the associative network. As here the update does not depend on the old state *v*(*t*), we may approximate transfer entropy by the regular transinformation or mutual information

(24) $$\begin{array}{l} T_{u\to v}=T\left(v\left(t+1\right);u\left(t\right)|v\left(t\right)\right)\approx T\left(F\left(u\left(t\right)\right);u\left(t\right)\right) \end{array}$$

(25) $$\begin{array}{l} =I\left(u\left(t\right)\right)-I\left(u\left(t\right)|F\left(u\left(t\right)\right)\right) \end{array}$$

(26) $$\begin{array}{l} =I\left(F\left(u\left(t\right)\right)\right)-I\left(F\left(u\left(t\right)\right)|u\left(t\right)\right) \end{array}$$

where *I*(*X*): = $-\underset{x}{\sum }p\left(x\right)\log p\left(x\right)$ is the Shannon information of a random variable *X*, and *I*(*X*|*Y*): = $-\underset{x,y}{\sum }p\left(x,y\right)\log p\left(x|y\right)$ the conditional information of *X* given *Y* (Shannon and Weaver, 1949; Cover and Thomas, 1991). Thus, up to normalization, transfer entropy $T_{u\to v}\approx T\left(F\left(u\left(t\right)\right);u\left(t\right)\right)=T\left({\hat{v}}^{\mu };{ũ}^{\mu }\right)$ has a very similar form as storage capacity *C*_*abs*_ in Equation (20). If *F*(*u*) is deterministic, the second term in Equation (26) vanishes and transfer entropy equals the output information *I*(*F*(*u*(*t*))) ≤ *I*(*u*(*t*)). If *F*(*u*) is also invertible, the second term in Equation (25) would vanish and *T*_*u*→*v*_ = *I*(*u*(*t*)) = *I*(*F*(*u*(*t*))) = *C*_*abs*_/*M*. However, in the associative memory application many input patterns are (ideally) mapped to one memory and *F*(*u*) is noninvertible and thus *T*_*u*→*v*_ = *I*(*F*(*u*(*t*))) < *I*(*u*(*t*)). Moreover, in more realistic cortex models *F* is also nondeterministic as *v*(*t*+1) will depend not only on activity *u*(*t*) from a single input area, but also on inputs from further cortical and subcortical areas as well as on numerous additional noise sources. Thus, in fact it will be *T*_*u*→*v*_ < *I*(*F*(*u*(*t*))).

Third, we can compare *T*_*u*→*v*_ to information storage capacity (Equation 20) by normalizing to single memory patterns,

(27) $$\begin{array}{l} \frac{C_{\text{abs}}}{M}=\frac{CPmn}{M}=T({\hat{v}}^{\mu };v^{\mu })=T(F(u(t);v^{\mu (u(t))}) \end{array}$$

(28) $$\begin{array}{l} =I\left(F\left(u\left(t\right)\right)\right)-I\left(F\left(u\left(t\right)\right)|v^{\mu \left(u\left(t\right)\right)}\right) \end{array}$$

where μ(*u*(*t*)) is a function determining the memory index of the input pattern *u*^μ(*u*(*t*))^ best matching the current input ũ = *u*(*t*). Thus, comparing Equation (26) to Equation (28) yields generally

(29) $$\begin{array}{l} T_{u\to v}-\frac{C_{\text{abs}}}{M}=I\left(F\left(u\left(t\right)\right)|v^{\mu \left(u\left(t\right)\right)}\right)-I\left(F\left(u\left(t\right)\right)|u\left(t\right)\right)\geq 0\;. \end{array}$$

where the bound is true as *v*^μ(*u*(*t*))^ is a deterministic function of *u*(*t*). In particular, for deterministic *F*, transfer entropy $T_{u\to v}=\frac{C_{\text{abs}}}{M}+I\left(F\left(u\left(t\right)\right)|v^{\mu \left(u\left(t\right)\right)}\right)$ typically exceeds normalized capacity $\frac{C_{\text{abs}}}{M}$, whereas equality follows for *I*(*F*(*u*(*t*))|*v*^μ(*u*(*t*))^) = *I*(*F*(*u*(*t*))|*u*(*t*)), for example, error-free retrieval with *F*(*u*(*t*)) = *v*^μ(*u*(*t*))^. Appendix A.4 shows that equality holds generally as well for nondeterministic propagation of activity (e.g., Equation 2 with N_*j*_≠0) if we assume that component retrieval errors occur independently with probabilities $q_{01}\;:=\text{pr}\left[{\hat{v}}_{j}^{\mu }=1|v_{j}^{\mu }=0\right]\approx \text{pr}\left[{\hat{v}}_{j}^{\mu }=1|v_{j}^{\mu }=0,ũ\right]=\text{pr}\left[{\hat{v}}_{j}^{\mu }=1|ũ\right]$ and *q*_10_: = $\text{pr}\left[{\hat{v}}_{j}^{\mu }=0|v_{j}^{\mu }=1\right]\approx \text{pr}\left[{\hat{v}}_{j}^{\mu }=0|v_{j}^{\mu }=1,ũ\right]=\text{pr}\left[{\hat{v}}_{j}^{\mu }=0|ũ\right]$ corresponding to the same (nondeterministic, i.i.d.) processing model as we have presumed in our capacity analysis (Figure 5A; see also Appendix A, Equations 42–43 or Equations 45–46 for Willshaw networks). Then normalizing transfer entropy TE and information capacity CN per output unit yields (see Equations 53, 38)

(30) $$\begin{array}{l} TE\;:=\frac{T_{u\to v}}{n}\overset{>}{\approx }T\left(q,q_{01},q_{10}\right)\approx CN\;:=\frac{C_{\text{abs}}}{Mn}=\frac{CPm}{M}\;. \end{array}$$

Thus, “effective connectivity” as measured by transfer entropy becomes (up to normalization) equivalent to the information storage capacity *C* of associative networks (see Equation 37 with Equation 38).

**Figure 6:**
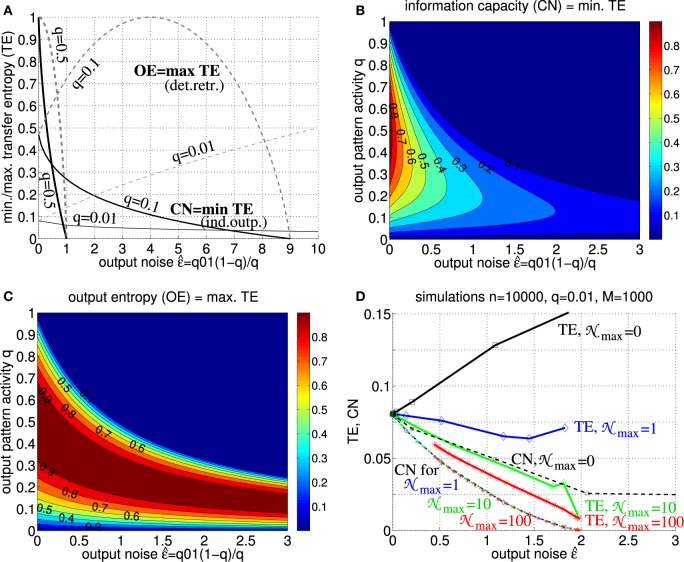
**Transfer entropy, output entropy and information capacity. (A)** Normalized transfer entropy (TE : = *T*_*u*→*v*_/*n*) is bounded by normalized information storage capacity (solid; CN: = *CPm*/*M* ≤ TE; see Equation 30 with Equation 38) and output entropy (dashed; OE$\;:=I\left({\hat{v}}_{j}^{\mu }\right)\geq$TE), where TE = OE for deterministic retrieval and TE = CN for non-deterministic retrieval with independent output noise (see text for details). The curves show TE,CN,OE as functions of output noise $\hat{\epsilon }=\left(1-q\right)q_{01}$ assuming only add noise $q_{01}=\text{pr}\left[{\hat{v}}_{j}=1|v_{j}=0\right]$ but no miss noise $q_{10}=\text{pr}\left[{\hat{v}}_{j}=0|v_{j}=1\right]=0$ (e.g., as it is the case for optimal “pattern part” retrieval; see Equation 46 in Appendix A.2). Different curves correspond to different fractions *q* of active units in a memory pattern (thick, medium, and thin lines correspond to *q* = 0.5, *q* = 0.1, and *q* = 0.01, respectively). **(B)** Contour plot of CN = min TE as function of output noise $\hat{\epsilon }$ and activity parameter *q* for *q*_10_ = 0. **(C)** Contour plot of OE=max TE as function of output noise $\hat{\epsilon }$ and activity parameter *q* for *q*_10_ = 0. **(D)** TE (thick solid) and CN (thin dashed) as functions of $\hat{\epsilon }$ for simulated retrieval (zero input noise $\tilde{\epsilon }=0$) in Willshaw networks of size *n* = 10, 000 storing *M* = 1000 cell assemblies of size *k* = 100 (*q* = 0.01) and increasing *P*_eff_ from 0 to 1 (markers correspond to *P*_eff_ = 0.001, 0.01, 0.1, 0.15, 0.2, …, 0.95, 1). Each data point corresponds to averaging over 10 networks each performing 10,000 retrievals of 100 memories (see Equations 51, 52). Different curves correspond to different levels of intrinsic noise N_*j*_ in output neurons *v*_*j*_ (see Equation 2; N_*j*_ uniformly distributed in [0;N_max_] for N_max_ = 0, 1, 10, 100 as indicated by black, blue, green, red lines). Note that, already for low noise levels, retrieval is non-deterministic such that TE becomes monotonic decreasing in $\hat{\epsilon }$ and, thus, similar or even equivalent to CN (and effectual connectivity *P*_eff_; see Figure 5B and Equation 49; cf. Figures 7, 8).

Figure 6 shows upper bounds *TE* ≤ *OE*: = $I\left(v_{j}^{\mu }\right)$ and lower bounds TE ≥ CN of transfer entropy as functions of output noise level $\hat{\epsilon }=qq_{10}+\left(1-q\right)q_{01}$ for different activities *q* of output patterns (cf., Equations 26, 29, 30). For low output noise ($\hat{\epsilon }\to 0$) both *T*_*u*→*v*_ and *C* approach the full information content of the stored memory set. In general both TE and CN are monotonic functions of $\hat{\epsilon }$ for relevant (sufficiently low) noise levels $\hat{\epsilon }$. While TE increases with $\hat{\epsilon }$ for deterministic retrieval (N_*j*_ = 0; cf. Equation 2), TE becomes a decreasing function of $\hat{\epsilon }$ already for low levels of intrinsic noise (N_*j*_ on the order of single synaptic inputs; see panel D). Similar decreases are obtained even without intrinsic noise, N_*j*_ = 0, if the target assembly *v*^μ^ receives (noisy) synaptic inputs from multiple cortical populations (data not shown; cf., Braitenberg and Schüz, 1991).

**Figure 7:**
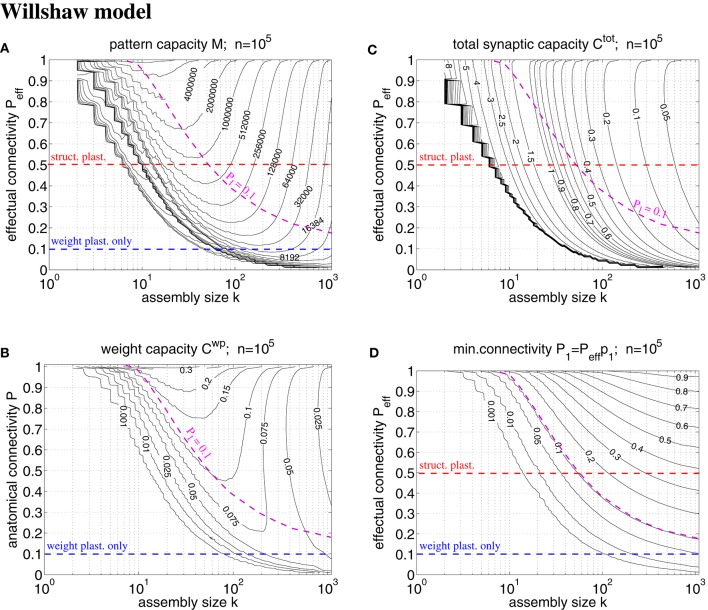
**Exact storage capacities for a finite Willshaw network having the size of a cortical macrocolumn (***n*** = 10^**5**^)**. **(A)** Contour plot of pattern capacity *M*_ϵ_ (maximal number of stored memories or cell assemblies) as a function of assembly size *k* (number of active units in a memory pattern) and effectual network connectivity *P*_eff_ assuming output noise level ϵ = 0.01 and no input noise (ũ = *u*^μ^). **(B)** Weight capacity $C_{\epsilon }^{\text{wp}}$ (in bit/synapse) corresponding to maximal *M*_ϵ_ in **(A)** for networks without structural plasticity. **(C)** Total storage capacity $C_{\epsilon }^{\text{tot}}$ (in bit/non-silent synapse) corresponding to maximal *M*_ϵ_ in **(A)** for networks with structural plasticity. Note that *C*^tot^ may increase even further if less than the maximum *M*_ϵ_ memories are stored (see text for details). **(D)** Minimal anatomical connectivity *P*_1_ = *p*_1_*P*_eff_ ≤ *P* required to achieve the data in **(A-C)**. Data computed as described in Appendix A.1. Red and blue dashed lines correspond to plausible values of *P*_eff_ for networks with and without structural plasticity (assuming *P* = 0.1, *P*_pot_ = 0.5). Note that only the area below the magenta dashed line (*P*_1_ = 0.1) is consistent with *P* = 0.1. Our exact data is in good agreement with earlier approximative data (Knoblauch et al., 2014, Figure 5) unless *k* is very small (e.g., *k* < 50).

**Figure 8:**
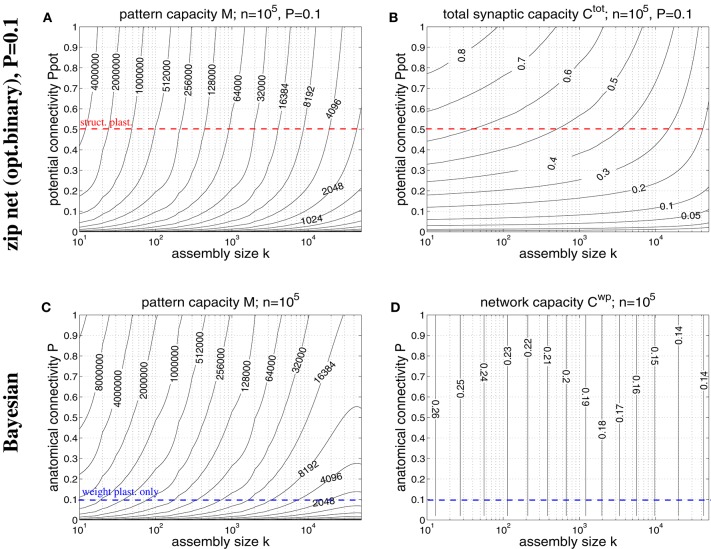
**Storage capacities for binary zip nets (A,B) and Bayesian neural networks (C,D) having the size of a cortical macrocolumn (***n*** = 10^**5**^)**. **(A)** Contour plot of the pattern capacity *M*_ϵ_ of an optimal binary zip net (employing the optimal covariance or homosynaptic learning rule; see Knoblauch, 2016) with *P* = *P*_1_ = 0.1 as a function of cell assembly size *k* and potential network connectivity *P*_pot_ (which is here an upper bound on the achievable effectual connectivity *P*_eff_). **(B)** Total storage capacity $C_{\epsilon }^{\text{tot}}$ for zip nets including structural plasticity for the setting of **(A)**. **(C)** Contour plot of the pattern capacity *M*_ϵ_ of an optimal Bayesian associative network (Knoblauch, 2011) without structural plasticity as a function of cell assembly size *k* and anatomical network connectivity *P*. **(D)** Weight capacity $C_{\epsilon }^{\text{wp}}$ for the Bayesian net for the setting of **(C)**. Other parameters are as assumed for Figure 7 (ϵ = 0.01, ũ = *u*^μ^). Data computed as described in Appendix A.3. Red and blue dashed lines correspond to plausible values for *P*_pot_ and *P*, respectively.

Our results thus show that, at least for realistic intrinsic noise and/or inter-columnar synaptic connectivity, transfer entropy *T*_*u*→*v*_ becomes equivalent to information capacity *C*. Because of the monotonic (or even linear) dependence of *C* on *P*_eff_ (see Figure 5B and Equation 49; cf. Figures 7, 8), transfer entropy is equivalent also to effectual connectivity *P*_eff_. Thus, we may interpret effectual connectivity *P*_eff_ as an essentially equivalent measure of “effective connectivity” as previously defined for functional brain imaging. Still, due to its anatomical definition, *P*_eff_ can only measure a *potential* causal interaction. For example, if both the synaptic connections from brain area *u* to *v* and the reverse connections from *v* to *u* have high *P*_eff_, we will not be able to infer the direction of information flow in a certain memory task unless we measure the actual neural activity.

### 3.2. Storage capacity of a macrocolumnar cortical network

A typical cortical macrocolumn comprises on the order of *n* = 10^5^ neurons below about 1 mm^2^ cortex surface, where the anatomical connectivity is about *P* = 0.1 and the potential connectivity about *P*_pot_ = 0.5 corresponding to a filling fraction *f*: = *P*/*P*_pot_ = 0.2 (Braitenberg and Schüz, 1991; Hellwig, 2000; Stepanyants et al., 2002). Sizes of cell assemblies have been estimated to be somewhere between 50 and 500 in entorhinal cortex (Waydo et al., 2006). Given these data we can try to estimate the number *M* of local cell assemblies or memories that can be stored in a macrocolumn (Sommer, 2000). In a previous work (Knoblauch et al., 2014, Figure 5) we have estimated the storage capacity for the Willshaw model (Figures 1, 4) by approximating dendritic potential distributions by Gaussians. However, this approximation can be off as, in particular, for sparse activity dendritic potentials can strongly deviate from Gaussians. We have therefore developed a method to compute the exact storage capacity for the Willshaw model storing random memories (see Appendix A). Figure 7 shows corresponding contour plots of pattern capacity *M*_ϵ_, weight capacity $C_{\epsilon }^{\text{wp}}$, total synaptic capacity $C_{\epsilon }^{\text{tot}}$, and the required minimal anatomical connectivity *P*_1_ (assuming that all silent synapses have been pruned in the end). We can make several observations: First, the exact results can significantly deviate from the approximations (cf., Knoblauch et al., 2014, Figure 5). In particular, for extremely sparse activity (*k* < 10) the Gaussian assumption seems violated and the true capacities are significantly lower than estimated previously. Still, for larger more realistic 50 < *k* < 500 the new data is in good agreement with the previous Gaussian estimates, and for even larger *k* > 500 the true capacities even slightly exceed the previous estimates. Second, the previous conclusions, therefore, largely hold: Without structural plasticity (*P*_eff_ = *P* = 0.1) the storage capacity would be generally very low and only a small number of memories could be stored. For very sparse *k* ≈ 50 not even a single memory could be stored and thus, the cell assembly hypothesis would be inconsistent with experimental estimates of *k*. Third, by contrast, networks including structural plasticity increasing *P*_eff_ from *P* = 0.1 to *P*_pot_ ≈ 0.5 can store many more memories: For example, for *k* = 50, the pattern capacity increases from *M* ≈ 0 to about *M* ≈ 800, 000. For *k* = 500, there is still an increase from *M* ≈ 13, 000 to *M* ≈ 45, 000. Fourth, correspondingly, networks without structural plasticity would have only a very small weight capacity *C*^wp^: For example, at *P*_eff_ = *P* = 0.1 it is *C*^wp^ ≈ 0bps for *k* ≤ 50 and still *C*^wp^ < 0.07bps for *k* = 500. Fifth, by contrast, networks with structural plasticity have a much higher total synaptic capacity *C*^tot^, i.e., they can store much more information per actual synapse and are therefore also much more energy-efficient, in particular for sparse activity: Although the very high values *C*^tot^ → log *n* are approached only for unrealistically low *k* and high *P*_eff_, they can still store *C*^tot^ ≈ 0.5bps for realistic *P*_eff_ = 0.5 and *k* = 50. This high value appears to decrease, however, to only *C*^tot^ ≈ 0.06bps for *k* = 500 which would suggest that, for relatively large cell assemblies with *k* = 500, a network without structural plasticity (at *P* = 0.1) would be more efficient than a network with structural plasticity (at *P*_eff_ = 0.5). However, as the Willshaw model is known to be sub-optimal for relatively large *k* ≫ log *n*, we will re-discuss this issue below for a more general network model. Sixth, another weakness of the Willshaw model is that the fraction $p_{1}\;:=1-{\left(1-\frac{k^{2}}{n^{2}}\right)}^{M}$ of 1-synapses is coupled both to cell assembly size *k* and number of stored memories *M* (due to the fixed synaptic threshold θ = 1, cf., Equation 1). Therefore, the residual (minimal) anatomical connectivity of a pruned network *P*_1_ = *p*_1_*P*_eff_ depends also on *k*,*M*, and we can obtain *P*_1_ ≈ *P* = 0.1 consistent with physiology only in a limited range of the *k-P*_eff_-planes of Figure 7. At least, physiological *k* ≈ 50 and *P*_eff_ ≈ 0.5 match physiological *P*_1_ = 0.1, whereas larger *k* ≫ 50 would require *P*_1_ being larger than the anatomical connectivity *P* = 0.1. As many cortical areas comprise significant fractions *P*_0_ > 0 of silent synapses we may as well allow for smaller *P*_1_ < *P* = 0.1 satisfying *P*_0_+*P*_1_ = *P* (where *C*^tot^ would become a measure only of energy efficiency, but no longer of space efficiency), but the very high values of *C*^tot^ ≫ 1 can generally be reached only for tiny fractions of 1-synapses.

In order to overcome some weaknesses of the Willshaw model we have recently proposed a novel network model (so called binary “zip nets”) where the fraction *p*_1_ of potentiated 1-synapses is no longer coupled to cell assembly size *k* and number *M* (Knoblauch, 2009b, 2010b, 2016). Instead, the model assumes that synaptic thresholds θ_*ij*_ (see Equation 1) are under homeostatic control to maintain a constant fractions *p*_1_ (or *P*_1_) of potentiated 1-synapses. We have shown for the limit *Mpq* → ∞ that this model can reach for *p*_1_ = 0.5 up to a “zip” factor ζ ≈ 0.64 almost the same high storage capacities *M*_ϵ_ and $C_{\epsilon }^{\text{wp}}$ as the optimal Bayesian neural network (Kononenko, 1989; Lansner and Ekeberg, 1989; Knoblauch, 2011), although requiring only binary synapses. Moreover, if compressed by structural plasticity, zip nets can also reach $C_{\epsilon }^{\text{tot}}\to \log n$ for *p*_1_ → 0, similar to the Willshaw model. As the Willshaw model is optimal only for extremely sparse activity (*k* ≤ log *n*) it is thus interesting to evaluate the performance gain of structural plasticity for physiological *k* using the zip net instead of the Willshaw model. Figure 8 shows data from evaluating storage capacity of a cortical macrocolumn of size *n* = 10^5^ both for the zip net model (upper panels) and the Bayesian model (lower panels), the latter being a benchmark for the optimal network without structural plasticity (Knoblauch, 2011). In order to compute the capacity of the zip net we have assumed physiological anatomical connectivity *P* = *P*_1_ = 0.1 where structural plasticity “moves” the $P_{1}n^{2}$ relevant 1-synapses to the most useful locations within the limits given by potential connectivity *P*_pot_ (as *P*_1_ is fixed, unlike to the Willshaw model, final *P*_eff_ after learning may be lower than *P*_pot_ in zip nets; see Appendix A.3 for methodological details). We can make the following observations: First, as expected, for high connectivity and very sparse activity (e.g., *k*≪100) the zip nets may perform worse than the Willshaw model (because the Willshaw model then performs close to the optimal Bayesian net). Second, for more physiological parameters *P*_pot_ ≤ 0.5, *k*≥50 the zip net can store significantly more memories than the Willshaw model, for example, for *P*_pot_ = 0.5 the zip net reaches *M* ≈ 1000000 for *k* = 50 and still *M* ≈ 120, 000 for *k* = 500. Third, also the total synaptic capacity *C*^tot^ is higher than for the Willshaw network, for example for *P*_pot_ = 0.5, it is *C*^tot^ ≈ 0.6 for *k* = 50 and still *C*^tot^ ≈ 0.5 for *k* = 500 (remember that the corresponding value for the Willshaw model required unphysiological *P*_1_ > 0.1). Fourth, although the Bayesian network can store significantly more memories *M* it has only a moderate storage capacity below *C*^wp^ = 0.25. In fact, for plausible cell assembly sizes, the binary synapses of the zip net with structural plasticity at *P* = 0.1 and *P*_pot_ = 0.5 achieve more than double the capacity of the optimal (but biologically implausible) Bayesian network with real-valued synapses at *P* = 0.1.

In summary, the new data confirms our previous conclusion that structural plasticity strongly increases space and energy efficiency of associative memory storage in neural networks under physiological conditions (Knoblauch et al., 2014).

### 3.3. Structural plasticity and the spacing effect

In previous works we have linked structural plasticity and cognitive effects like retrograde amnesia, absence of catastrophic forgetting, and the spacing effect (Knoblauch, 2009a; Knoblauch et al., 2014). Here we focus on a more detailed analysis of the spacing effect that learning is most efficient if learning is distributed in time (Ebbinghaus, 1885; Crowder, 1976; Greene, 1989). For example, learning a list of vocabularies in two sessions each lasting 10 min is more efficient than learning in a single session of 20 min. We have explained this effect by slow ongoing structural plasticity and fast synaptic weight plasticity: Thus, spaced learning is useful because during the (long) time gaps between two (or more) learning sessions structural plasticity can grow many novel synapses that are potentially useful for storing new memories and that can quickly be potentiated and consolidated by synaptic weight plasticity during the (brief) learning sessions (Knoblauch et al., 2014, Section 7.3).

Appendix B.2 develops a simplified theory of the spacing effect that is based on model variant B of a potential synapse (which can more easily be analyzed than model A; see Figure 3) and the concept and methods proposed in Section 2.3. In particular, with (Equations 73–75) we can easily compute the temporal evolution of effectual connectivity *P*_eff_(*t*) for arbitrary rehearsal sequences of a novel set of memories to be learned. As output noise $\hat{\epsilon }$ is a decreasing function of *P*_eff_ (see Figure 5B), we can use *P*_eff_ as a measure of retrieval performance.

**Figure 9:**
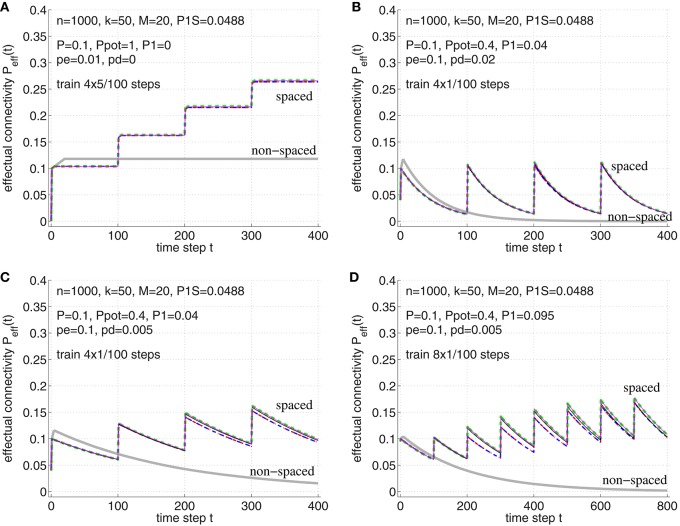
**Verification of the theoretical analyses of the spacing effect in Section B.2 in Appendix**. Each curve shows effectual connectivity *P*_eff_ over time for different network and learning parameters. Thin solid lines correspond to simulation experiments of synapse model A (magenta; see Figure 3A) and synapse model B (black; see Figure 3B), where both variants assume that at most one synapse can connect a neuron pair (𝔭(1) = 1). Green dashed lines correspond to the theory of synapse model A in Appendix B.1 (see Equations 54–56). Blue dash-dotted lines correspond to the theory of synapse model B in Appendix B.2 (see Equations 71–72) and, virtually identical, red-dashed lines correspond to the final theory of model B (see Equations 73–75). For comparison, thick light-gray lines correspond to non-spaced rehearsal of the same total duration as the spaced rehearsal sessions (using model A). **(A)** Spaced rehearsal of a set of *M* = 20 memories at times *t* = 0−4, 100−104, 200−204, and 300−304. Each memory had *k* = *l* = 50 active units out of *m* = *n* = 1000 neurons corresponding to a consolidation load *P*_1*S*_ ≈ 0.0488. Further we used anatomical connectivity *P* = 0.1, potential connectivity *P*_pot_ = 1, initial fraction of consolidated synapses of *P*_1_ = 0 and *p*_*e*|1_ = *p*_*d*|1_ = 0, *p*_*c*|*s* = *s*_. In each simulation step a fraction *p*_*e*_: = *p*_*e*|0_ = 0.01 of untagged silent synapses was replaced by new synapses at other locations, but there was no deconsolidation *p*_*d*_: = *p*_*d*|0_ = 0. **(B)** Similar parameters as before, but *P*_pot_ = 0.4, *P*_1_ = 0.04, *p*_*e*_ = 0.1, and *p*_*d*_ = 0.02. Memories were rehearsed for a single time step *t* = 0, *t* = 100, *t* = 200, and *t* = 300. **(C)** Similar parameters as for panel B, but smaller *p*_*d*_ = 0.05. **(D)** Similar parameters as for panel C, but larger *P*_1_ = 0.095, i.e., 95 percent of real synapses are initially consolidated. Rehearsal times were *t* = 0, 100, 200, …, 700. Note that the theoretical curves for model A closely match the experimental curves (magenta vs. green). The theory for model B is still reasonably good (black vs. blue/red), although panel D shows some deviations to the simulation experiments. Such deviations may be due to the small number of unstable silent synapses (*P*_1_ near *P*). In any case, synapse models A and B behave very similar.

To illustrate the effect of spaced vs. non-spaced rehearsal (or consolidation) on *P*_eff_, and to verify the theory in Appendix B.2, Figure 9 shows the temporal evolution of *P*_eff_(*t*) for different models and synapse parameters. It can be seen that for high potential connectivity *P*_pot_ ≈ 1 and low deconsolidation probability *p*_*d*|*s*_ ≈ 0 the spacing effect is most pronounced and the network easily realizes high-performance long-term memory (with high *P*_eff_; see panel A). Larger *p*_*d*|0_ > 0 is plausible to model short-term memory, whereas realizing long-term memory would then require repeated consolidation steps (panels B–D). Significant spacing effects are visible for any parameter set. Comparing the microscopic simulations of both synapse models from Figure 3 to the macroscopic simulations using the methods of Section 2.3 and Appendix B.2, it can be seen that all model and simulation variants behave qualitatively and quantitatively very similar. This justifies to use the theory of Appendix B.2 in the following analysis of recent psychological experiments exploring the spacing effect.

**Figure 10:**
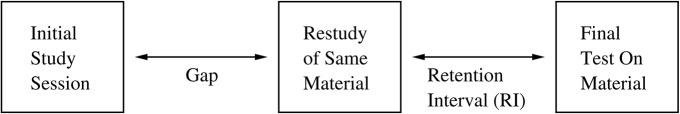
**Structure of a typical study of spacing effects on learning**. Study episodes are separated by a varying gap, and the final study episode and test are separated by a fixed retention interval. Figure modified from Cepeda et al. (2008).

For example, Cepeda et al. (2008) describe an internet-based learning experiment investigating the spacing effect over longer time intervals of more than a year (up to 455 days). The structure of the experiment followed Figure 10. The subjects had to learn a set of facts in an initial study session. After a gap interval (0–105 days) without any learning the subjects restudied the same material. After a retention interval (RI; 7–350 days) there was the final test.

These experiments showed that the final recall performance depends both on the gap and the RI showing the following characteristics: First, for any gap duration, recall performance decline as a function of RI in a negatively accelerated fashion, which corresponds to the familiar “forgetting curve.” Second, for any RI greater than zero, an increase in study gap causes recall to first increase and then decrease. Third, as RI increases, the optimal gap increases, whereas that ratio of optimal gap to RI declines. The following shows that our simple associative memory model based on structural plasticity can explain most of these characteristics.

**Figure 11:**
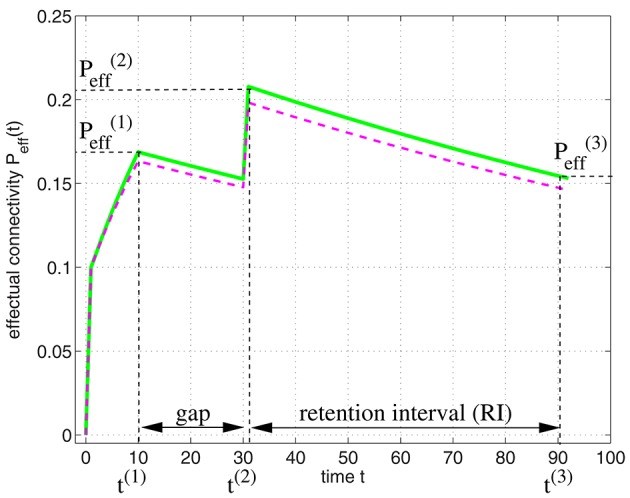
**Modeling the spacing effect experiment of Cepeda et al. (2008) as illustrated by Figure 10**. Curves show effectual connectivity *P*_eff_ as function of time *t* according to the theory of synapse model A (green solid; Figure 3A; see Appendix B.1) and synapse model B (magenta dashed; Figure 3B; see Equations 73–75). In an initial study session, facts are learned until some desired performance level $P_{\text{eff}}^{\left(1\right)}$ is reached at time *t*^(1)^ = 10. After a gap the facts are rehearsed briefly at time *t*^(2)^ = 30 reaching a performance equivalent to $P_{\text{eff}}^{\left(2\right)}$. After the retention interval at time *t*^(3)^ = 90 performance has decreased corresponding to an effectual connectivity $P_{\text{eff}}^{\left(3\right)}$. Parameters were *P* = 0.1, *P*_pot_ = 0.4, *P*_1_ = 0, *P*_1*S*_ = 0.1, *p*_*c*|*s*_ = *s*, *p*_*e*|0_ = 0.1, *p*_*d*|0_ = 0.005, and *p*_*e*|1_ = *p*_*d*|1_ = 0.

It is straight-forward to model the experiments of Cepeda et al. (2008) by applying our model of structural plasticity and synaptic consolidation. Figure 11 illustrates *P*_eff_(*t*) for a learning protocol as employed in the experiments: In an initial study session facts are learned until time *t*^(1)^ when some desired performance level $P_{\text{eff}}^{\left(1\right)}$ is reached. After a gap the facts are rehearsed briefly at time *t*^(2)^ reaching a performance equivalent to $P_{\text{eff}}^{\left(2\right)}$. After the retention interval at time *t*^(3)^ performance still corresponds to an effectual connectivity $P_{\text{eff}}^{\left(3\right)}$.

Similar to Cepeda et al. (2008), we want to optimize the gap duration in order to maximize $P_{\text{eff}}^{\left(3\right)}$ for a given retention interval RI. After the second rehearsal at time *t*^(2)^, *P*_eff_ decays exponentially by a fixed factor 1−*p*_*d*|0_ per time step (Equation 74). Therefore, $P_{\text{eff}}^{\left(3\right)}=P_{\text{eff}}^{\left(2\right)}{\left(1-p_{d|0}\right)}^{t^{\left(3\right)}-t^{\left(2\right)}}$ is a function of $P_{\text{eff}}^{\left(2\right)}$ that decreases with the retention interval length *t*^(3)^−*t*^(2)^. We can therefore equivalently maximize $P_{\text{eff}}^{\left(2\right)}$ with respect to the gap length Δ*t*: = *t*^(2)^−*t*^(1)^. For *p*_*c*|*s*_ = *s*, *p*_*e*|1_ = *p*_*d*|1_ = 0, a good approximation of $P_{\text{eff}}^{\left(2\right)}$ follows from Equation (73),

(31) $$\begin{array}{l} P_{\text{eff}}^{\left(2\right)}\approx \frac{\begin{array}{c} PP_{\text{pot}}+\left[\left(P_{\text{pot}}-P\right)P_{\text{eff}}^{\left(1\right)}-P_{\text{pot}}P_{1}^{\left(t1\right)}\right]{\left(1-p_{d|0}\right)}^{\Delta t}\\ -P_{\text{pot}}\left(P-P_{1}^{\left(t1\right)}\right){\left(1-p_{e|0}\right)}^{\Delta t} \end{array}}{P_{\text{pot}}-P_{1}^{\left(t1\right)}{\left(1-p_{d|0}\right)}^{\Delta t}-\left[P-P_{1}^{\left(t1\right)}\right]{\left(1-p_{e|0}\right)}^{\Delta t}}\;, \end{array}$$

where $P_{1}^{\left(t1\right)}\;:=P_{1}^{\left(t0\right)}\left(1-P_{1S}\right){\left(1-p_{d|0}\right)}^{t^{\left(1\right)}}+P_{1S}P_{\text{eff}}^{\left(1\right)}$ with $P_{1}^{\left(t0\right)}$ denoting the initial fraction of consolidated synapses at time 0.^3^ Since $P_{\text{eff}}^{\left(2\right)}$ does not depend on the RI we can already see that the optimal gap interval Δ*t* depends on the RI neither (which contrasts with the experiments reporting that optimal Δ*t* increases with RI). Optimizing Δ*t* yields the optimality criterion (see Appendix B.3)

(32) $$\begin{array}{l} \frac{P_{\text{eff}}^{\left(1\right)}-P_{1}^{\left(t1\right)}}{P-P_{1}^{\left(t1\right)}}+\left(\alpha -1\right)\frac{P_{\text{eff}}^{\left(1\right)}}{P_{\text{pot}}}x^{\alpha }-\alpha x^{\alpha -1}=0\;. \end{array}$$

with

(33) $$\begin{array}{l} x:={\left(1-p_{d|0}\right)}^{\Delta t}=e^{\Delta t\ln\left(1-p_{d|0}\right)}\Leftrightarrow \;\Delta t=\frac{\ln\;x}{\ln\left(1-p_{d|0}\right)} \end{array}$$

(34) $$\begin{array}{l} \alpha :=\frac{\ln\left(1-p_{e|0}\right)}{\ln\left(1-p_{d|0}\right)}\;, \end{array}$$

which can easily be evaluated using standard Newton-type numerical methods. Note that Equation (32) can be used to link neuroanatomical and neurophysiological to psychological data. For example, given the optimal gap Δ*t*_opt_ from psychological experiments, Equation (32) gives a constraint on the remaining network and learning parameters. Alternatively, we can solve Equation (32) to determine the optimal gap Δ*t*_opt_ given the remaining parameters.

**Figure 12:**
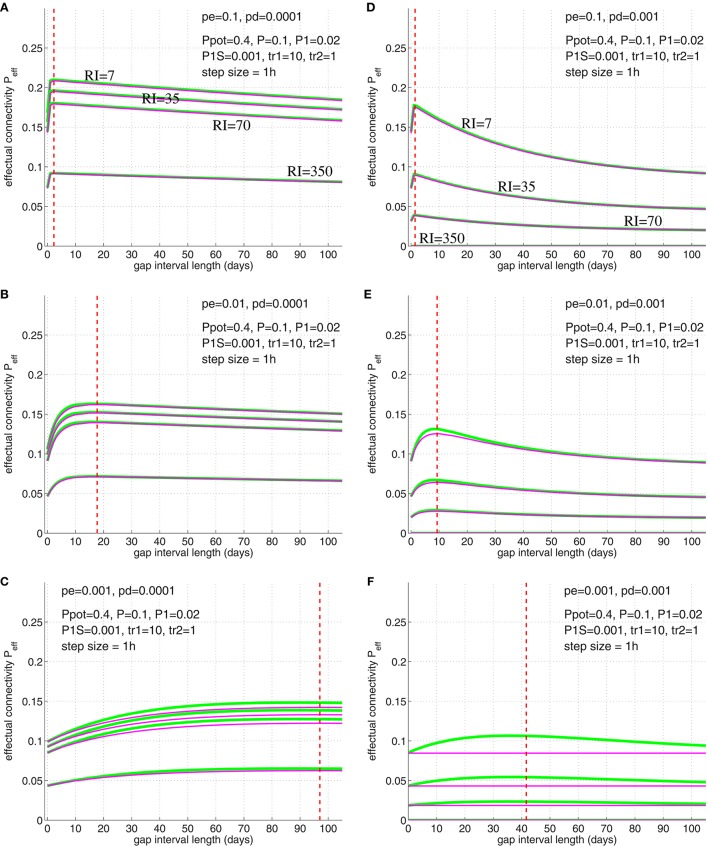
**Simulation of the spacing effect described by Cepeda et al. (2008, Figure 3) using synapse model variant A (green lines) and B (magenta lines; see Figure 3)**. Each curve shows final effectual connectivity $P_{\text{eff}}=P_{\text{eff}}^{\left(3\right)}$ as a function of rehearsal gap Δ*t* for different retention intervals (RI = 7, 35, 70, 350 days) assuming an experimental setting as in illustrated in Figures 10, 11. Initially, memory facts were rehearsed for tr1=10 time steps (1 time step = 1 h). After the gap, memory facts were rehearsed again for a single time step (tr2 = 1). Finally, after RI steps the resulting effectual connectivity was tested. Red dashed lines indicate optimal gap interval length for synapse model **B** as computed from solving Equation (32). Different panels correspond to different synapse parameters *p*_*e*|0_ and *p*_*d*|0_: Elimination probabilities are *p*_*e*|0_ = 0.1 (top panels **A,D**), *p*_*e*|0_ = 0.01 (middle panels **B,E**), and *p*_*e*|0_ = 0.001 (bottom panels **C,F**). Deconsolidation probabilities are *p*_*d*|0_ = 0.0001 (left panels **A–C**) and *p*_*d*|0_ = 0.001 (right panels **D–F**). Remaining model parameters are described in the main text.

We have verified Equation (32) by simulations illustrated in Figure 12 (compare simulation data to Cepeda et al., 2008, Figure 3). For these simulations we chose physiologically plausible model parameters: Similarly as before we used *P*_pot_ = 0.4 (Stepanyants et al., 2002; DePaola et al., 2006), *P* = 0.1 (Braitenberg and Schüz, 1991; Hellwig, 2000). Further, we used $P_{1}^{\left(t0\right)}=0.02$ as neurophysiological experiments investigating two-state properties of synapses suggest that about 20% of synapses are in the “up” state (Petersen et al., 1998; O'Connor et al., 2005)^4^. Then we chose a small consolidation load *P*_1*S*_ = 0.001 assuming that the small set of novel facts is negligible compared to the presumably large set of older memories. As before, we assumed *p*_*g*_ in homeostatic balance to maintain a constant anatomical connectivity *P*(*t*) (Equation 69) and binary consolidation signals *s* = *S*_*ij*_∈{0, 1} with *p*_*c*|*s*_ = *s* and *p*_*d*|1_ = *p*_*e*|1_ = 0 for any synapse *ij*. For the remaining learning parameters *p*_*e*|0_ and *p*_*d*|0_ we have chosen several combinations to test their relevance for fitting the model to the observed data.

The simulation results of Figure 12 imply the following conclusions: First, the simulations show that the optimal gap determined by Equation (32) closely matches the simulation results, for both synapse models (Figure 3). Second, for fixed deconsolidation *p*_*d*|0_, larger *p*_*e*|0_ implies smaller optimal gaps Δ*t*_opt_. Thus, faster synaptic turnover implies smaller optimal gaps. Third, for fixed turnover *p*_*e*|0_, larger *p*_*d*|0_ implies smaller Δ*t*_opt_. Thus, faster deconsolidation implies also smaller optimal gaps. Fourth, together this means that faster (weight and structural) plasticity implies smaller optimal gaps. Fifth, although model variants A and B (Figure 3) behave very similar for most parameters settings, they can differ significantly for some parameter combinations. For example, for *p*_*e*|0_ = *p*_*d*|0_ = 0.001 (panel F) the peak in *P*_eff_ of model A is more than a third larger than the peak of model B. In fact, there the curve of model B is almost flat. Still, even here, the optimal gap interval length is very similar for the two models. An obvious reason why model A sometimes performs better than model B is that deconsolidation of a synapse in model A does not necessarily imply elimination as in model B (see Figure 3). Sixth, our simple model already satisfies two of the three characteristics of the spacing effect mentioned above: Both the forgetting effect and the existence of an optimal time gap can be observed in a wide parameter range. Best fits to the experimental data occurred for *p*_*e*|0_ = 0.01 and *p*_*d*|0_ = 0.0002 (between parameters of panels B,C; data not shown). Last, however, our simple model cannot reproduce the third characteristic: As argued above, the optimal gap interval length Δ*t*_opt_ does not depend on the retention interval RI. This is in contrast to the experiments of Cepeda et al. (2008) reporting that Δ*t*_opt_ increases with RI.

Nevertheless, we have shown in some preliminary simulations that a slight extension of the model can easily resolve the latter discrepancy (Knoblauch, 2010a): By mixing two populations of synapses having different plasticity parameters corresponding to a small and large optimal gap (or fast and slow plasticity), respectively, it is possible to obtain a dependence of optimal spacing as in the experiments.

## 4. Discussion

In this theoretical work we have identified roles of structural plasticity and effectual connectivity *P*_eff_ for network performance, measuring brain connectivity, and optimizing learning protocols. Analyzing how many cell assemblies or memories can be stored in a cortical macrocolumn (of size 1 mm^3^), we find a strong dependence of storage capacity on *P*_eff_ and cell assembly size *k* (see Figures 7, 8). We find that, without structural plasticity, when cell assemblies would have a connectivity close to the low anatomical connectivity *P* ≈ 0.1, only a small number of relatively large cell assemblies could be stably stored (Latham and Nirenberg, 2004; Aviel et al., 2005) and, correspondingly, retrieval would not be energy efficient (Attwell and Laughlin, 2001; Laughlin and Sejnowski, 2003; Lennie, 2003; Knoblauch et al., 2010; Knoblauch, 2016). It thus appears that storing and efficiently retrieving a large number of small cell assemblies as observed in some areas of the medial temporal lobe (Waydo et al., 2006) would require structural plasticity increasing *P*_eff_ from the low anatomical level toward the much larger level of potential connectivity *P*_pot_ ≈ 0.5 (Stepanyants et al., 2002). Similarly, our model predicts ongoing structural plasticity for any cortical area that exhibits sparse neural activity and high capacity.

Moreover, we have shown a close relation between our definition of effectual connectivity *P*_eff_ and previous measures of functional brain connectivity. While the latter, for example transfer entropy, are solely based on correlations between neural activity in cortical areas (Schreiber, 2000), our definition of *P*_eff_ as the fraction of realized required synapses has also a clear anatomical basis (Figure 2). Via the link of memory channel capacity *C*(*P*_eff_) used to measure storage capacity of a neural network, we have shown that *P*_eff_ is basically an equivalent measure of functional connectivity as transfer entropy. By this, it may become possible to establish an anatomically grounded link between structural plasticity and functional connectivity. For example, this could enable predictions on which cortical areas exhibit strong ongoing structural plasticity during certain cognitive tasks.

Further, as one example linking cognitive phenomena to its potential anatomical basis, we have more closely investigated the spacing effect that learning becomes more efficient if rehearsal is distributed to multiple sessions (Crowder, 1976; Greene, 1989; Cepeda et al., 2008). In previous works we have already shown that the spacing effect can easily be explained by structural plasticity and that, therefore, structural plasticity may be the common physiological basis of various forms of the spacing effect (Knoblauch, 2009a; Knoblauch et al., 2014). Here we have extended these results to explain some recent long-term memory experiments investigating the optimal time gap between two learning sessions (Cepeda et al., 2008). For a given retention interval, our model, if fitted to neuroanatomical data, can easily explain the profile of the psychological data, in particular, the existence of an optimal gap that maximizes memory retention. It is even possible to analyze this profile, linking the optimal gap to parameters of the synapse model, in particular, the rate of deconsolidation *p*_*d*|0_ and elimination *p*_*e*|0_. Our results show that small optimal gaps correspond to fast structural and weight plasticity with a high synaptic turnover rate *p*_*e*|0_ and relative large *p*_*d*|0_ with a high forgetting rate, whereas large gaps correspond to slow plasticity processes. This result has two implications: First, it may be used to explain the remaining discrepancy that in the psychological data the time gap depends on the retention interval, whereas in our model it does not: As preliminary simulations indicate, the experimental data could be reproduced by mixing (at least) two synapse populations with different sets of parameters, where they could be both within the same cortical area (stable vs. unstable synapses; cf., Holtmaat and Svoboda, 2009) or distributed to different areas (e.g., fast plasticity in the medial temporal lobe, and slower plasticity in neocortical areas). Moreover, as the temporal profile of optimal learning depends on parameters of structural plasticity, it may become possible in future experiments to link behavioral data on memory performance to physiological data on structural plasticity in cortical areas where these memories are finally stored.

Although we have concentrated on analyzing one-step retrieval in feed-forward networks, our results apply as well to recurrent networks and iterative retrieval (Hopfield, 1982; Schwenker et al., 1996; Sommer and Palm, 1999): Obviously, all results on the temporal evolution of *P*_eff_ (including the results on the spacing effect) depend only on synapses having proper access to consolidation signals *S*_*ij*_ by either repeated rehearsal or memory replay, and therefore hold independently of network and retrieval type. However, linking *P*_eff_ to output noise (Equation 3) needs to assume a particular retrieval procedure. At least one-step retrieval is known to be almost equivalent for both feedforward and recurrent networks yielding almost identical output noise and pattern capacity *M*_ϵ_ (Knoblauch, 2008). Estimating retrieved information for pattern completion in auto-associative recurrent networks, however, requires to subtract the information already provided by the input patterns ũ^μ^. Here information storage capacity *C* is maximal if ũ^μ^ contains half of the one-entries (or information) of the original pattern *u*^μ^, which leads to factor 1/2 and 1/4 decreases of *M* and *C* compared to hetero-association (cf., Equations 48, 49 for λ = 1/2; Palm and Sommer, 1992). Nevertheless, up to such scaling, our results demonstrating *C* increasing with *P*_eff_ are still valid. Similarly, our capacity analyses of *M*_ϵ_ and *C*_ϵ_ can also be applied to iterative retrieval by requiring that the one-step output noise level ϵ is smaller than the initial input noise $\tilde{\epsilon }$. As typically output noise $\hat{\epsilon }$ steeply decreases with input noise $\tilde{\epsilon }$ (cf. Equation 45), additional retrieval steps will drive $\hat{\epsilon }$ toward zero, with activity quickly converging to the memory attractor.

Our theory depends on the assumption that potential connectivity *P*_pot_ is significantly larger than anatomical connectivity *P*. This assumption may be challenged by experimental findings suggesting that cortical neuron pairs are either unconnected or have multiple (e.g., 4 or 5) instead of single synapses (Fares and Stepanyants, 2009) and the corresponding theoretical works to explain these findings (Deger et al., 2012; Fauth et al., 2015b). For example, Fauth et al. (2015a) predict that narrow distributions of synapse numbers around 4 or 5 follow from a regulatory interaction between synaptic and structural plasticity, where connections having a smaller synapse number cannot stably exist. If true this would mean that most potential synapses could never become stable actual synapses because the majority of potentially connected neuron pairs have less than 4 potential synapses (e.g., see Fares and Stepanyants, 2009, Figure 1). As a consequence, actual *P*_pot_ would be significantly lower than assumed in our work, perhaps only slightly larger than *P*, strongly limiting a possible increase of effectual connectivity *P*_eff_ by structural plasticity. On the other hand, the data of Fares and Stepanyants (2009) are based only on neuron pairs having very low distances (< 50μm), whereas our model rather applies to cortical macrocolumns where most neuron pairs have much larger distances. Thus, unlike Fauth et al. (2015a), our theory of structural plasticity increasing effectual connectivity and synaptic storage efficiency predicts that neuron pairs within a macrocolumn should typically be connected by a much smaller synapse number (e.g., 1 or perhaps 2).

## Author contributions

Conceived, designed, and performed experiments: AK. Analyzed the data: AK, FS. Contributed simulation/analysis tools: AK. Wrote the paper: AK, FS.

## Funding

FS was supported by INTEL, the Kavli Foundation and the National Science Foundation (grants 0855272, 1219212, 1516527).

### Conflict of interest statement

The authors declare that the research was conducted in the absence of any commercial or financial relationships that could be construed as a potential conflict of interest.

## Footnotes

^1^More precisely, this means the presence of *at least one synapse* connecting the first to the second neuron. This definition is motivated by simplifications employed by many theories for judging how many memories can be stored. These simplifications include, in particular, (1) point neurons neglecting dendritic compartments and non-linearities, and (2) ideal weight plasticity such that any desired synaptic strength can be realized. Then having two synapses with strength 1 would be equivalent to a single synapse with strength 2. The definition is further justified by experimental findings that the number of actual synapses per connection is narrowly distributed around small positive values (Fares and Stepanyants, 2009; Deger et al., 2012).

^2^The general case considers delay vectors (*u*(*t*), *u*(*t*−1), …, *u*(*t*−*K*+1) and (*v*(*t*), *v*(*t*−1), …, *v*(*t*−*L*+1)) instead of *u*(*t*) and *v*(*t*).

^3^Note that a constant (instead of decaying) “background” consolidation *P*_1_ can be modeled, for example, by using $P_{1}^{\left(t0\right)}=0$ and then excluding the initially consolidated synapses from further simulation. This means to simulate a network with anatomical connectivity $P^{\prime }=P-P_{1}$, potential connectivity $P_{\text{pot}}^{\prime }=P_{\text{pot}}-P_{1}$, no initial consolidation with $P_{1}^{\prime }=0$, and otherwise same parameters as the original network. Then the effectual connectivity can be computed from $P_{1}^{\left(1\right)}={P_{1}^{\left(1\right)}}^{\prime }+P_{1S}P_{1}$ using Equation (18) where ${P_{1}^{\left(1\right)}}^{\prime }mn$ is obtained from the simulation.

^4^It may be more realistic that the total number of “up”-synapses is kept constant by homeostatic processes (i.e., *P*_1_/*P* = 0.2). However, here we were more interested in verifying our theory which assumes exponential decay of “up”-synapses. To account for homeostasis with constant *P*_1_ one may proceed as described in footnote 3. Nevertheless, the qualitative behavior of the model does not strongly depend on *P*_1_ or $P_{1}^{\left(t0\right)}$ unless their values being close to *P* which would strongly impair learning.
