# Supplementary material for: Structural Plasticity, Effectual Connectivity, and Memory in Cortex
Source: Front Neuroanat. 2016 Jun 16;10:63. doi: 10.3389/fnana.2016.00063 (PMC4909771; doi:10.3389/fnana.2016.00063)
Supplement: Supplementary file 1 [file MathematicalAppendix.pdf]

MATHEMATICAL APPENDIX

A STORAGE CAPACITY OF ASSOCIATIVE NETWORKS

The general approach for estimating storage capacity of a memory device (such as the Willshaw network illustrated by Fig. 1) is to compute retrieval error probabilities as a function of stored patterns M. Then the capacity is the maximal M such that retrieval errors remain below some critical bound. For example, we may express retrieval errors in terms of *output noise*

$$\hat{\epsilon} := \frac{(n-l)q_{01} + lq_{10}}{l} \tag{35}$$

defined as the mean Hamming distance $d_H(\hat{v}, v^\mu) := \sum_{j=1}^{n} |\hat{v}_j - v_j^\mu|$ between retrieval output $\hat{v}$ and the original memory v^μ normalized to the content memory activity l. Here $q_{01} := \mathrm{pr}[\hat{v}_j = 1 | v_j^\mu = 0]$ and $q_{10} := \mathrm{pr}[\hat{v}_j = 0 | v_j^\mu = 1]$ are component output error probabilities (similarly, we define input error probabilities $p_{01} := \mathrm{pr}[\tilde{u}_i = 1 | u_i^\mu = 0]$ and $p_{10} := \mathrm{pr}[\tilde{u}_i = 0 | u_i^\mu = 1]$). Thus, we can define *pattern capacity* M_ϵ (absolute number of memories) and the *synaptic information storage capacity* C_ϵ (in bit per synapse) at output noise level ϵ,

$$M_\epsilon := \max\{M : \hat{\epsilon} \leq \epsilon\} \tag{36}$$

$$C_\epsilon := \frac{M_\epsilon T(l/n, q_{01}, q_{10})}{Pm} \tag{37}$$

where T is the transinformation (or mutual information) when transmitting the memory component v_j^μ over a binary channel and receiving $\hat{v}_j$. It is

$$\begin{aligned} T(q, q_{01}, q_{10}) &:= I_{\hat{v}}(q, q_{01}, q_{10}) - I_{\hat{v}|v}(q, q_{01}, q_{10}) \\ &\approx I(q) := -q\,\mathrm{ld}q - (1-q)\mathrm{ld}(1-q) \approx -q\,\mathrm{ld}q \end{aligned} \tag{38}$$

where $I(q)$ is the Shannon information (or entropy) of a memory component v_j^μ (for $q := l/n$), $I_{\hat{v}}(q, q_{01}, q_{10}) := I(q(1-q_{10}) + (1-q)q_{01})$ is the information of an output component $\hat{v}_j$, and $I_{\hat{v}|v^\mu}(q, q_{01}, q_{10}) := qI(q_{10}) + (1-q)I(q_{01})$ is the information of $\hat{v}_j$ given v_j^μ. The first and second approximations are true for small $\epsilon \ll 1$ and $q \ll 1$, respectively. For networks without structural plasticity we call C_ϵ also "network capacity" or "weight capacity" and write C_ϵ^{wp} to indicate that storage involves only plasticity of synaptic weights. If networks include both weight plasticity and structural plasticity we call C_ϵ also "total synaptic capacity" and write $C_\epsilon^{\mathrm{tot}}$ (in some previous works we have written C^{tot} also as C^S; cf., **Knoblauch et al.**, 2010; **Knoblauch**, 2011, 2016).

A.1 EXACT CAPACITY FOR THE WILLSHAW MODEL

We first compute the storage capacity for the Willshaw model (eq. 1 for fixed $\theta_{ij} = 1$) without structural plasticity. Exact error probabilities q_{01} and q_{10} for one step retrieval (eq. 2 with $\mathcal{N}_j = 0$) can be computed from the Willshaw-Palm probability distribution (**Knoblauch**, 2008; **Sommer and Palm**, 1999; **Buckingham and Willshaw**, 1992). Let W be the weight matrix of the fully connected Willshaw network with $P = 1$ after storing M associations between binary random vectors u^μ (having k out of m one-entries) and v^μ (having l out of n one-entries). Then stimulating with a random query $\tilde{u}^*$ that is unrelated

to the stored memories and has exactly z out of m one-entries implies dendritic potentials distributed as

$$
\begin{aligned}
p_{\mathrm{Ph}}(x;k,l,m,n,M,\tilde{p}_1,1,z) &:= \mathrm{pr}[\sum_{i=1}^{m} W_{ij}\tilde{u}_i^* = x] \\
&= \binom{z}{x}\sum_{s=0}^{x}(-1)^s\binom{x}{s}(1-\tilde{p}_1)^{s+z-x}(1-\frac{l}{n}(1-B(m,k,s+z-x)))^M
\end{aligned} \tag{39}
$$

for $0 \leq x \leq z$ (and $p_{\mathrm{Ph}}(x) = 0$ otherwise), $B(m,k,s) := \binom{m-k}{s}/\binom{m}{s} = \prod_{i=0}^{s-1}(m-k-i)/(m-i) = B(m,s,k)$, and $\tilde{p}_1$ being the fraction of noisy one-entries in the weight matrix (e.g., $\tilde{p}_1 = 0$ for W as in eq. 1). For the general case of diluted networks with anatomical connectivity $P \leq 1$ we enforce $W_{ij} = 0$ with probability $1-P$ corresponding to non-existing synapses. Thus, generalizing eq. 39 yields

$$
p_{\mathrm{Ph}}(x;k,l,m,n,M,\tilde{p}_1,P,z) = \sum_{x'=x}^{z} p_{\mathrm{Ph}}(x';k,l,m,n,M,\tilde{p}_1,1,z)p_B(x;x',P) \tag{40}
$$

$$
\approx \sum_{i=0}^{M} p_B(i;M,l/n)p_B(x;z,P(1-(1-\tilde{p}_1)(1-k/m)^i)) \tag{41}
$$

where $p_B(x;N,P) := \binom{N}{x}P^x(1-P)^{N-x}$ is the binomial probability. The approximation becomes accurate and computationally cheaper for $M \ll z$. Now assume the general case that the query pattern $\tilde{u}$ has c "correct" one-entries in common with an address vector u^μ and additionally f "false" one-entries. Assuming that all neurons have the same firing threshold Θ, the retrieval error probabilities compute

$$
q_{01}(\Theta) = \sum_{x=\Theta}^{c+f} p_{\mathrm{Ph}}(x;k,l,m,n,M-1,\tilde{p}_1,P,c+f) \tag{42}
$$

$$
q_{10}(\Theta) = \sum_{x=0}^{\Theta-1}\sum_{x'=x}^{c+f} p_{\mathrm{Ph}}(x'-c;k,l,m,n,M,\tilde{p}_1,1,f)p_B(x;x',P)
$$

$$
= \sum_{x=0}^{\Theta-1}\sum_{x_f=0}^{f} p_{\mathrm{Ph}}(x_f;k,l,m,n,M,\tilde{p}_1,P,f)p_B(x-x_f,c,P) \tag{43}
$$

We can optimize Θ in order to minimize output noise $\hat{\epsilon}$ (eq. 35) yielding the final error probabilities and, thus, pattern capacity eq. 36. For larger networks evaluation of the formulas requires high computing precision and is computational expensive. We have evaluated the formulas with the $TBigFloat$ data type of the Felix++ neural simulation tool using mantissas with 1000 bit precision. To compute capacities for the Willshaw model *with* structural plasticity where all memories are stored at the same effectual connectivity $P_{\mathrm{eff}} \leq P_{\mathrm{pot}}$, we can simply replace P in eqs. 42,43 by P_{eff} as argued in **Knoblauch et al.** (2014).

A.2 ASYMPTOTIC CAPACITY OF THE WILLSHAW MODEL

Under further simplifying assumptions (for example, that each neuron can optimize firing thresholds individually; **Buckingham and Willshaw**, 1993) it is possible for the Willshaw model to derive more convenient expressions for asymptotic storage capacity in closed form. Slightly extending the results of **Knoblauch et al.** (2014, eqs.13-15,27) for general hetero-association, it is for sparse activity ($k, l \ll$

m, n) and input patterns $\tilde{u}$ having a fraction $\lambda := 1 - p_{10}$ of correct one-entries, but zero add-noise ("pattern part retrieval" with $p_{01} = 0$),

$$p_1 = 1 - (1 - \frac{kl}{mn})^M \tag{44}$$

$$q_{01} \approx \sum_{c'=0}^{\lambda k} p_B(c'; c, P_{\text{eff}}) p_1^{c'} = (1 - P_{\text{eff}}(1 - p_1))^{\lambda k} . \tag{45}$$

$$q_{10} = 0 \tag{46}$$

$$p_{1\epsilon} \approx H\left(\frac{\left(\frac{\epsilon l}{n-l}\right)^{\frac{1}{\lambda k}} - (1 - P_{\text{eff}})}{P_{\text{eff}}}\right) \in (0, 1) \tag{47}$$

$$M_\epsilon \approx \lambda \frac{mn}{l \ln \frac{n-l}{\epsilon l}} \ln(1 - P_{\text{eff}}(1 - p_{1\epsilon})) \ln(1 - p_{1\epsilon}) \tag{48}$$

$$C_\epsilon^{\text{wp}} \approx \lambda \frac{\text{ld}(1 - P_{\text{eff}}(1 - p_{1\epsilon})) \ln(1 - p_{1\epsilon})}{P_{\text{eff}}} \eta \leq 0.69 \tag{49}$$

$$C_\epsilon^{\text{tot}} = C_\epsilon^{\text{wp}} / p_{1\epsilon} \tag{50}$$

where p_1 is the fraction of potentiated synapses (in a corresponding network without structural plasticity), $p_{1\epsilon}$ is maximal p_1 at output noise level ϵ, H is the Heaviside function, and $\eta \approx (1 + (\ln \epsilon)/\ln(l/n))^{-1} \to 1$ for large networks (**Knoblauch et al.**, 2010, eq. 3.11).

A.3 CAPACITY OF BAYESIAN AND BINARY ZIP NETWORKS

For associative networks with optimal Bayesian learning and binary zip nets we cannot compute exact capacities, but only approximations that become exact in the limit of large networks with $Mpq \to \infty$. For Bayesian neural networks without structural plasticity the pattern capacity M_ϵ can be computed as in **Knoblauch** (2011, eq. 2.33). Similarly, for binary zip nets using a linear learning rule (eq. 1) without structural plasticity, M_ϵ can be computed as in **Knoblauch** (2016, eq. 4.3) using the binary zip factor ζ given by **Knoblauch** (2016, eq. 5.8). For the data of Fig. 8 we have further assumed zero input noise ($\tilde{\epsilon} = 0$) and for the zip net an optimal linear learning rule (e.g., homosynaptic rule with $R(1, 1) = 1 - q, R(1, 0) = -q, R(0, 1) = R(0, 0) = 0$). For estimating the storage capacity of the zip net with structural plasticity (Fig. 8) we have made the following assumptions: First, all M memories can be reactivated (by replay or rehearsal) for an arbitrary long time such that the Pmn synapses will all be potentiated and placed at the most useful locations constrained by potential connectivity. Therefore, the resulting pattern capacity M_ϵ of the structurally plastic network with anatomical connectivity P and potential connectivity P_{pot} equals the pattern capacity of a zip net without structural plasticity having an anatomical connectivity of P_{pot} and a fraction $p_1 := \min(P/P_{\text{pot}}, 0.5)$ of potentiated 1-synapses (here p_1 is chosen as large as possible up to the optimum $p_1 = 0.5$, cf., **Knoblauch**, 2016, eq. 5.9).

A.4 COMPARISON BETWEEN TRANSFER ENTROPY AND STORAGE CAPACITY

Assuming i.i.d. output units v_j (as in the previous capacity analyses), the normalized transfer entropy per output unit follows from eq. 26 as

$$TE := \frac{T_{u \to v}}{n} \approx \frac{I(\hat{v}) - I(\hat{v}|\tilde{u})}{n} \approx I(\hat{v}_j) - I(\hat{v}_j|\tilde{u}) = I_{\hat{v}}(q, q_{01}, q_{10}) - I(\hat{v}_j|\tilde{u}) \tag{51}$$

where $I_{\hat{v}}(q, q_{01}, q_{10})$ is as defined below eq. 38, where we have skipped indices μ for brevity.

To account for such nondeterministic propagation of activity $v(t+1) = F(u(t))$ or $\hat{v} = F(\tilde{u})$ during retrieval (cf., eq. 2) we assume $p(\hat{v}_j|\tilde{u}) \approx p(\hat{v}_j|v_j)$ as argued above eq. 30. This assumption is justified in particular for "pattern part retrieval" with optimal threshold control as presumed in the asymptotic analysis of app. A.2, where $q_{10} = 0$ (eq. 46): Then $p(\hat{v}_j|\tilde{u}) = 1$ for all "correct" output units j with $v_j = 1$ and additionally some "false" units j with $v_j = 0$ that are determined by $\tilde{u}$. Similarly, $p(\hat{v}_j|v_j) = 1$ for $v_j = 1$, but $p(\hat{v}_j|v_j) = q_{01}$ for all units j with $v_j = 0$. Thus, our approximation corresponds to the same average output noise, but assumes that output errors $\hat{v}_j \neq v_j$ occur nondeterministically in each retrieval, even for identical inputs $\tilde{u}$. [5] Thus, for $p(\hat{v}_j|\tilde{u}) \approx p(\hat{v}_j|v_j)$ with $v_j := v(\tilde{u})_j$ we obtain

$$
\begin{aligned}
I(\hat{v}_j|\tilde{u}) &= -\sum_{\tilde{u},\hat{v}_j} p(\tilde{u},\hat{v}_j)\log p(\hat{v}_j|\tilde{u}) = -\sum_{\tilde{u},\hat{v}_j} p(\tilde{u})p(\hat{v}_j|\tilde{u})\log p(\hat{v}_j|\tilde{u}) \\
&\approx -\sum_{\tilde{u},\hat{v}_j} p(\tilde{u})p(\hat{v}_j|v(\tilde{u})_j)\log p(\hat{v}_j|v(\tilde{u})_j) = -\sum_{v_j}\sum_{\tilde{u}:v(\tilde{u})_j=v_j} p(\tilde{u})\sum_{\hat{v}_j} p(\hat{v}_j|v_j)\log p(\hat{v}_j|v_j) \\
&= -\sum_{v_j} p(v_j)\sum_{\hat{v}_j} p(\hat{v}_j|v_j)\log p(\hat{v}_j|v_j) = -\sum_{v_j,\hat{v}_j} p(\hat{v}_j,v_j)\log p(\hat{v}_j|v_j) = I(\hat{v}_j|v_j)
\end{aligned}
\tag{52}
$$

where $I(\hat{v}_j|v_j) = I_{\hat{v}|v}(q, q_{01}, q_{10})$ as defined below eq. 38.Thus eq. 51 approximates eq. 38,

$$
TE := \frac{T_{u\to v}}{n} \approx I_{\hat{v}}(q, q_{01}, q_{10}) - I_{\hat{v}|v}(q, q_{01}, q_{10}) =: T(q, q_{01}, q_{10})\,. \tag{53}
$$

B ANALYSIS AND SIMPLIFIED MODEL OF STRUCTURAL PLASTICITY

B.1 MACROSCOPIC SIMULATION OF GENERAL NETWORKS OF POTENTIAL SYNAPSES

For further analyses and efficient simulation, let us derive a simplified system of six differential equations from eqs. 8-18 making the following assumptions: First, let P_{pot} be a given potential connectivity where at most one synapse connects a neuron pair, i.e., $\mathfrak{p}(\mathfrak{n}) = 1$ for $\mathfrak{n} = 1$ and zero otherwise. Second, let $S_{ij} \in \{0, 1\}$ be a given binary consolidation signal defining only two groups of potential synapses where the index $s \in \{0, 1\}$ denotes potential synapses with $S_{ij} = s$. Third, let $R(t) \in \{0, 1\}$ indicate rehearsal or memory replay, i.e., $R(t) = 1$ iff at time t there is rehearsal or replay of the memories defined by the consolidation signal S_{ij}. Then the macroscopic group-specific state variables (eqs. 11-12 with eqs. 8-10) simplify to

$$
P_1^{(s)}(t) = (1 - p_{d|R(t)s})P_1^{(s)}(t-1) + p_{c|R(t)s}P_0^{(s)}(t-1) \tag{54}
$$

$$
P_0^{(s)}(t) = (1 - p_{c|R(t)s} - p_{e|R(t)s})P_0^{(s)}(t-1) + p_{d|R(t)s}P_1^{(s)}(t-1) + p_g P_\pi^{(s)}(t-1) \tag{55}
$$

$$
P_\pi^{(s)}(t) = (1 - p_g)P_\pi^{(s)}(t-1) + p_{e|R(t)s}P_0^{(s)}(t-1)\,. \tag{56}
$$

[5] We consider such non-deterministic retrieval more realistic for cortex models, because any cortical areas v typically receives input from multiple cortical and subcortical sites instead of a single area u. These additional inputs (among other noise-related factors) would transform the deterministic membrane potential x in eq. 2 to a random variable and, thus, retrieval would become non-deterministic.

and eqs. 14-18 rewrite

$$\begin{aligned}
P_{\mathrm{state}}(t) &= P^{(0)}_{\mathrm{state}}(t) + P^{(1)}_{\mathrm{state}}(t) \quad \text{for state} \in \{\pi, 0, 1\} && (57)\\
P(t) &= P_0(t) + P_1(t) && (58)\\
P_{\mathrm{pot}} &= P_\pi(t) + P_0(t) + P_1(t) && (59)\\
P_{1S} &= (P^{(1)}_\pi(t) + P^{(1)}_0(t) + P^{(1)}_1(t))/P_{\mathrm{pot}} && (60)\\
P_{\mathrm{eff}}(t) &= P^{(1)}_1(t)/P_{1S}\ . && (61)
\end{aligned}$$

For given $P_1(0)$ one may assume initial parameters $P^{(1)}_1(0) = P_{1S}P_1(0)$ (such that $P_{\mathrm{eff}}(0) = P_1(0)$), $P^{(1)}_0(0) = P_{1S}P_0(0)$ with $P_0(0) = P - P_1(0)$, and $P^{(1)}_\pi(0) = P_{1S}P_\pi(0)$ with $P_\pi(0) = P_{\mathrm{pot}} - P$.

Eqs. 54-56 can be rewritten as a system of non-linear differential equations if we interpret the transition probabilities (e.g., p_g) as rates (e.g., $r_g := p_g/dt$) and assuming vanishing time step size $dt \to 0$,

$$\begin{aligned}
\frac{d}{dt}P^{(s)}_1 &= -r_{d|Rs}P^{(s)}_1 + r_{c|Rs}P^{(s)}_0 && (62)\\
\frac{d}{dt}P^{(s)}_0 &= -(r_{c|Rs} + r_{e|Rs})P^{(s)}_0 + r_{d|Rs}P^{(s)}_1 + r_g P^{(s)}_\pi && (63)\\
\frac{d}{dt}P^{(s)}_\pi &= -r_g P^{(s)}_\pi + r_{e|Rs}P^{(s)}_0 && (64)\\
& && (65)
\end{aligned}$$

For constant $R(t)$ any initial state configuration will converge for $t \to \infty$ to a unique steady-state solution obtained from $d/dtP^{(s)}_{\mathrm{state}} = 0$,

$$\begin{aligned}
P^{(s),\infty}_1(t) &= \frac{sP_{1S} + (1-s)(1-P_{1S}}{1 + \frac{r_{d|Rs}}{r_{c|Rs}}(1 + \frac{r_{e|Rs}}{r_g})}P_{\mathrm{pot}} && (66)\\
P^{(s),\infty}_0(t) &= \frac{r_{d|Rs}}{r_{c|Rs}}P^{(s)}_1 && (67)\\
P^{(s),\infty}_\pi(t) &= \frac{r_{d|Rs}r_e}{r_{c|Rs}r_g}P^{(s)}_1 && (68)
\end{aligned}$$

where eq. 66 follows with eq. 60.

If generation and elimination of synapses are in homeostatic balance then p_g and r_g can be replaced by time-dependent variables

$$p_g(t) = \frac{p_{e|0}P^{(0)}_0(t-1) + p_{e|R(t)}P^{(1)}_0(t-1)}{P_\pi} \quad \text{and} \quad \mathrm{r_g(t)} = \frac{\mathrm{r_{e|0}P^{(0)}_0(t) + r_{e|R(t)}P^{(1)}_0(t)}}{\mathrm{P_\pi}}\ , \qquad (69)$$

respectively, implying constant P and P_π. Then the network can be described by only three independent equations for $P^{(1)}_1$, $P^{(0)}_1$, $P^{(1)}_0$, for example, whereas $P^{(1)}_\pi = P_{\mathrm{pot}}P_{1S} - P^{(1)}_1 - P^{(1)}_0$, $P^{(0)}_\pi = P_\pi - P^{(1)}_\pi$, $P^{(0)}_0 = P - P^{(1)}_1 - P^{(0)}_1 - P^{(1)}_0$.

B.2 APPLICATION FOR ANALYZING THE SPACING EFFECT

Here we adapt and extend the theory developed in **Knoblauch et al.** (2014, eq. 11,app. I.2) describing the increase of effectual connectivity

$$\begin{aligned} P_{\rm eff}(t) &\approx P_{\rm pot} - (P_{\rm pot} - P) / \prod_{t_1=1}^{t-t_0-1} \left(1 + p_{e|0} \frac{P - (1-P_{1S})(1-p_{d|0})^{t_1} P_1(t_0) - P_{1S} P_{\rm eff}(t_1)}{P_{\rm pot} - P} \right) \\ &\approx P_{\rm pot} - (P_{\rm pot} - P) e^{-(t-t_0-1)\ln\left(1+p_{e|0}\frac{P-P_1(0)}{P_{\rm pot}-P}\right)} \end{aligned} \tag{70}$$

during ongoing consolidation at time $t = t_0 + 1, t_0 + 2, \ldots$ for analysis of the spacing effect. As there, we assume large networks ($m, n \gg 1$), at most a single synapse per neuron pair, binary $s \in \{0, 1\}$, memories being independent of network structure, $p_{c|s} = s$, $p_{d|1} = p_{e|1} = 0$, and constant $P(t)$. To keep the analysis as simple as possible we consider potential synapses according to model variant B to approximate the more realistic variant A (see Fig. 3A,B). Let $P_{\rm eff}(t_1)$ be the effectual connectivity at time t_1. At time $t_1 + \Delta t$, but not in the interim time between t_1 and $t_1 + \Delta t$, there is a single replay or rehearsal of the memory items to be stored. Then the effectual connectivity after that rehearsal is

$$P_{\rm eff}(t_1 + \Delta t) = P_{\rm eff}(t_1)((1-p_{d|0})^{\Delta t} + (1-(1-p_{d|0})^{\Delta t})G/L) + (P_{\rm pot} - P_{\rm eff}(t_1))G/L \,, \tag{71}$$

where G is the number of actual synapses at time t_1 that get eliminated (at least once) until time $t_1 + \Delta t$, and L is the number of potential locations where G new synapses could be generated (to maintain constant P). The intuition of the formula is that a required synapse is an actual synapse at time $t_1 + \Delta t$ if (1) it was already an actual synapse at time t_1 and is not eliminated until time $t_1 + \Delta t$ or it is eliminated but regenerated at $t_1 + \Delta t$, or (2) it was not an actual synapse at t_1 but generated at $t_1 + \Delta t$. On average it is $G/mn = P_0(t_1)(1-(1-p_{e|0})^{\Delta t}) + P_1(t_1)(1-(1-p_{d|0})^{\Delta t})$ and $L/mn = G + P_{\rm pot} - P$, and therefore

$$\frac{1}{G/L} \approx 1 + \frac{P_{\rm pot} - P}{(P - P_1(t_1))(1-(1-p_{e|0})^{\Delta t}) + P_1(t_1)(1-(1-p_{d|0})^{\Delta t})} \,. \tag{72}$$

With this eq. 71 simplifies to

$$P_{\rm eff}(t_1+\Delta t) \approx \frac{P P_{\rm pot} + [(P_{\rm pot} - P)P_{\rm eff}(t_1) - P_{\rm pot} P_1(t_1)](1-p_{d|0})^{\Delta t} - P_{\rm pot}(P - P_1(t_1))(1-p_{e|0})^{\Delta t}}{P_{\rm pot} - P_1(t_1)(1-p_{d|0})^{\Delta t} - [P - P_1(t_1)](1-p_{e|0})^{\Delta t}} \tag{73}$$

whereas in time intervals without rehearsal, $t_1 < t < t_1 + \Delta t$, effectual connectivity decays according to

$$P_{\rm eff}(t) = P_{\rm eff}(t_1)(1-p_{d|0})^{t-t_1} \,. \tag{74}$$

Finally, the fraction of consolidated synapses at time t is

$$P_1(t) = P_1(t_0)(1-P_{1S})(1-p_{d|0})^{t-t_0} + P_{1S} P_{\rm eff}(t) \,, \tag{75}$$

where $P_1(t_0)$ at initial time t_0 (before the first rehearsal) corresponds to the initially consolidated synapses that are unrelated to the new memory traces to be stored. [6] The fraction of unconsolidated silent synapses is always $P_0(t) = P - P_1(t)$ for any t (eq. 15). Note that these formulae generalize eq. 70 including intervals without rehearsal or replay (Δt). Thus, with the initial conditions $P_{\rm eff}(t_0) = P_1(t_0)$ and $P_{\rm eff}(t_0 + 1) = P$

[6] Alternatively, we may assume constant P_1 because (1) the load $P_{1S} \approx 0$ of the few novel memories may be negligible compared to the much larger P_1 from background consolidation of older memories; and/or (2) P_1 may be regulated itself to a constant value (e.g., $P_1 = 0.2P$; cf., **Petersen et al.**, 1998; **O'Connor et al.**, 2005; **Knoblauch**, 2016).

(from eq. 70), they allow an efficient forward computation of effectual connectivity P_{eff} for arbitrary rehearsal sequences $T_R = (t_{R1}, t_{R2}, \ldots)$ of a *single* memory set (where $t_{R1} < t_{R2} < \ldots$ are rehearsal times):

- For a rehearsal time $t = t_{Ri}$ we can compute $P_{\text{eff}}(t)$ from eq. 73 using $t_1 := t_{Ri-1}$ and $\Delta t := t_{Ri} - t_{Ri-1}$.
- For an intermediary time step t with $t_{Ri} < t < t_{Ri+1}$ we can compute $P_{\text{eff}}(t)$ from eq. 74 using $t_1 := t_{Ri}$.

Figure 9 illustrates that, for most parameters, results derived from eqs. 73-75 are virtually identical to microscopic simulations of potential synapses (of both model variants A and B; cf., Fig. 3) and also to the macroscopic simulation method proposed in section 2.3. Further details can be found in a technical report (**Knoblauch**, 2010c, app. B.2)

B.3 OPTIMAL SPACING OF LEARNING

To compute optimal time gaps between rehearsal sessions in section 3.3, we optimize eq. 31 with respect to the gap Δt. Eq. 31 has the form

$$P_{\text{eff}}^{(2)}(x) \approx \frac{a + bx + cx^{\alpha}}{u + vx + wx^{\alpha}} \tag{76}$$

with $a := PP_{\text{pot}}$, $b := (P_{\text{pot}} - P)P_{\text{eff}}^{(1)} - P_{\text{pot}}P_1^{(t1)}$, $c := -P_{\text{pot}}(P - P_1^{(t1)})$, $u := P_{\text{pot}}$, $v := -P_1^{(t1)}$, $w := -(P - P_1^{(t1)})$, $x := (1 - p_{d|0})^{\Delta t}$, and $\alpha := \ln(1 - p_{e|0})/\ln(1 - p_{d|0})$. As Δt occurs only in x, we have to maximize $P_{\text{eff}}^{(2)}(x)$ with respect to x. The derivative is proportional to

$$\frac{d}{dx}P_{\text{eff}}^{(2)}(x) \sim (b + \alpha c x^{\alpha-1})(u + vx + wx^{\alpha}) - (a + bx + cx^{\alpha})(v + \alpha w x^{\alpha-1}) \tag{77}$$

The optimum occurs for zero derivative or, equivalently,

$$(bu - av) + x^{\alpha}(1 - \alpha)(bw - cv) + x^{\alpha-1}\alpha(cu - aw) = 0 \tag{78}$$

It is

$$\begin{aligned} bu - av &= P_{\text{pot}}(P_{\text{pot}} - P)P_{\text{eff}}^{(1)} - P_{\text{pot}}^2 P_1^{(t1)} + PP_{\text{pot}}P_1^{(t1)} \\ &= P_{\text{pot}}(P_{\text{pot}} - P)(P_{\text{eff}}^{(1)} - P_1^{(t1)}) \end{aligned} \tag{79}$$

$$\begin{aligned} bw - cv &= -(P_{\text{pot}} - P)P_{\text{eff}}^{(1)}(P - P_1^{(t1)}) + P_{\text{pot}}P_1^{(t1)}(P - P_1^{(t1)}) - P_{\text{pot}}(P - P_1^{(t1)})P_1^{(t1)} \\ &= -(P_{\text{pot}} - P)(P - P_1^{(t1)})P_{\text{eff}}^{(1)} \end{aligned} \tag{80}$$

$$\begin{aligned} cu - aw &= -P_{\text{pot}}^2(P - P_1^{(t1)}) + PP_{\text{pot}}(P - P_1^{(t1)}) \\ &= -(P - P_1^{(t1)})P_{\text{pot}}(P_{\text{pot}} - P) \end{aligned} \tag{81}$$

and therefore eq. 78 writes

$$P_{\text{pot}}(P_{\text{eff}}^{(1)} - P_1^{(t1)}) + (\alpha - 1)(P - P_1^{(t1)})P_{\text{eff}}^{(1)}x^{\alpha} - \alpha(P - P_1^{(t1)})P_{\text{pot}}x^{\alpha-1} = 0 \tag{82}$$

and we finally obtain the optimality criterion eq. 32.
